# Evolution of neuronal cell classes and types in the vertebrate retina

**DOI:** 10.1038/s41586-023-06638-9

**Published:** 2023-12-13

**Authors:** Joshua Hahn, Aboozar Monavarfeshani, Mu Qiao, Allison H. Kao, Yvonne Kölsch, Ayush Kumar, Vincent P. Kunze, Ashley M. Rasys, Rose Richardson, Joseph B. Wekselblatt, Herwig Baier, Robert J. Lucas, Wei Li, Markus Meister, Joshua T. Trachtenberg, Wenjun Yan, Yi-Rong Peng, Joshua R. Sanes, Karthik Shekhar

**Affiliations:** 1 Department of Chemical and Biomolecular Engineering, University of California, Berkeley, Berkeley, CA USA; 2 Department of Cellular and Molecular Biology, Center for Brain Science, Harvard University, Cambridge, MA USA; 3 Division of Biology and Biological Engineering, California Institute of Technology, Pasadena, CA USA; 4 Max Planck Institute for Biological Intelligence, Martinsried, Germany; 5 Retinal Neurophysiology Section, National Eye Institute, National Institutes of Health, Bethesda, MD USA; 6 Department of Cellular Biology, University of Georgia, Athens, GA USA; 7 Division of Neuroscience and Centre for Biological Timing, Faculty of Biology Medicine and Health, University of Manchester, Manchester, UK; 8 Division of Chemistry and Chemical Engineering, California Institute of Technology, Pasadena, CA USA; 9 Department of Neurobiology, David Geffen School of Medicine at UCLA, Los Angeles, CA USA; 10 Department of Ophthalmology, Stein Eye Institute, UCLA David Geffen School of Medicine, Los Angeles, CA USA; 11 Helen Wills Neuroscience Institute,Vision Science Graduate Group, University of California, Berkeley, Berkeley, CA USA; 12 Biological Systems and Engineering Division, Lawrence Berkeley National Laboratory, Berkeley, CA USA; 13 Center for Computational Biology, Biophysics Graduate Group, University of California, Berkeley, Berkeley, CA USA; 14 California Institute of Quantitative Biosciences (QB3), University of California, Berkeley, Berkeley, CA USA; 15 Present Address: LinkedIn, Mountain View, CA USA

**Keywords:** Retina, Classification and taxonomy, Taxonomy

## Abstract

The basic plan of the retina is conserved across vertebrates, yet species differ profoundly in their visual needs^1^. Retinal cell types may have evolved to accommodate these varied needs, but this has not been systematically studied. Here we generated and integrated single-cell transcriptomic atlases of the retina from 17 species: humans, two non-human primates, four rodents, three ungulates, opossum, ferret, tree shrew, a bird, a reptile, a teleost fish and a lamprey. We found high molecular conservation of the six retinal cell classes (photoreceptors, horizontal cells, bipolar cells, amacrine cells, retinal ganglion cells (RGCs) and Müller glia), with transcriptomic variation across species related to evolutionary distance. Major subclasses were also conserved, whereas variation among cell types within classes or subclasses was more pronounced. However, an integrative analysis revealed that numerous cell types are shared across species, based on conserved gene expression programmes that are likely to trace back to an early ancestral vertebrate. The degree of variation among cell types increased from the outer retina (photoreceptors) to the inner retina (RGCs), suggesting that evolution acts preferentially to shape the retinal output. Finally, we identified rodent orthologues of midget RGCs, which comprise more than 80% of RGCs in the human retina, subserve high-acuity vision, and were previously believed to be restricted to primates^2^. By contrast, the mouse orthologues have large receptive fields and comprise around 2% of mouse RGCs. Projections of both primate and mouse orthologous types are overrepresented in the thalamus, which supplies the primary visual cortex. We suggest that midget RGCs are not primate innovations, but are descendants of evolutionarily ancient types that decreased in size and increased in number as primates evolved, thereby facilitating high visual acuity and increased cortical processing of visual information.

## Abstract (web summary)

Single-cell and single-nucleus transcriptomic analysis of retina from 17 vertebrate species shows high conservation of retinal cell types and suggests that midget retinal ganglion cells in primates evolved from orthologous cells in ancestral mammals.

## Main

The ability to assess gene conservation among species has been of great value in multiple ways. It has revealed the evolutionary history of specific genes, highlighted crucial developmental and functional pathways, informed strategies for rational in vivo manipulations and helped guide choices of animal models that mimic human diseases^3,4^. Comparative genomics was enabled by advances in DNA sequencing, as well as statistical methodologies for sequence alignment and phylogenetic inference^5^. Advances in high-throughput single-cell RNA sequencing (scRNA-seq) and single-nucleus RNA sequencing (snRNA-seq) have enabled related activity focused on determining the extent to which cell types, the functional units of complex tissues^6,7^, are conserved among species. Analysing patterns of cell-type conservation across phylogeny can serve as a conceptual foundation for reconstructing the evolution of cell types and identifying conserved developmental programmes^8–10^.

**Fig. 1:**
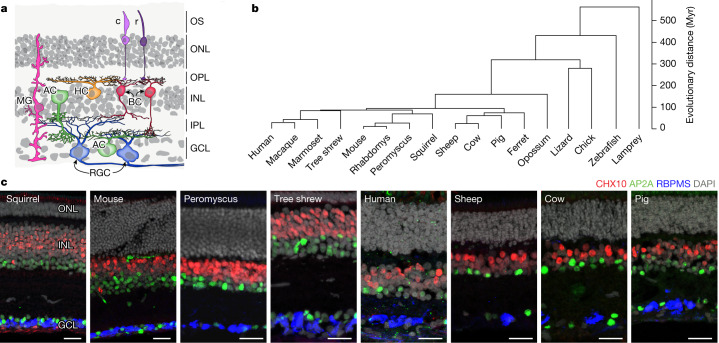
Conserved retinal structure across vertebrates. **a**, Cartoon of a section through a vertebrate retina showing the arrangement of its six major cell classes: photoreceptors (including rods (r) and cones (c)), horizontal cells (HC), bipolar cells (BC), amacrine cells (AC), retinal ganglion cells (RGC) and Müller glia (MG). The outer segments of rods and cones (OS), outer nuclear layer (ONL), inner nuclear layer (INL) and ganglion cell layer (GCL)—which contain cell somata—are indicated, as are the outer (synaptic) layer (OPL) and inner plexiform layer (IPL). **b**, Phylogeny of the 17 vertebrate species analysed in this work. The scale bar on the right indicates estimated divergence time. **c**, Sections from retinas of eight species immunostained for RBPMS (a pan-RGC marker), CHX10 (also known as VSX2) (a pan-bipolar cell marker) and AP2A (also known as TFAP2A) (a pan-amacrine cell marker) and stained with the nuclear stain DAPI. Scale bars, 25 µm. Figures are representative of images from three retinas.

The neural retina, the portion of the brain that resides in the back of the eye, is well-suited for this type of analysis. It is arguably as complex as any other part of the brain, but its compactness and accessibility facilitate detailed investigations of structure and function^11^. Moreover, unlike other brain regions (for example, the cerebral cortex), the basic structural blueprint of the retina is highly conserved among vertebrates^1^. The retina contains five neuronal classes—photoreceptors, horizontal cells, bipolar cells, amacrine cells and retinal ganglion cells (RGCs)—and a resident glial class called Müller glia^12^. The cell somata are arranged in three nuclear layers separated by two plexiform (synaptic) layers (Fig. 1a) with information flowing through them in a defined direction: photoreceptors in the outer nuclear layer sense light and transmit visually evoked signals to interneurons in the inner nuclear layer; the interneurons (horizontal cells, bipolar cells and amacrine cells) process the information and supply it to RGCs in the innermost layer; and the RGCs send axons through the optic nerve to visual centres in the brain. Several of the neuronal classes can be subdivided into subclasses, and all classes comprise multiple types that differ in morphology, physiology, connectivity and molecular composition^6,11–14^. The specificity of connections between interneuronal and RGC types endows many RGC types with selective responsiveness to small subsets of visual features such as edges, directional motion and chromaticity^14,15^. As a result of neural computations in the retina, the optic nerve transmits a set of parallel representations of the visual scene to the rest of the brain for further processing^16,17^.

Despite these conserved features, vertebrate species differ greatly in their visual needs^1^. Some species are diurnal, others are nocturnal; some are terrestrial, others are aquatic; and some mainly hunt, whereas others forage for colourful fruits. It is likely that variations in retinal cell types across species emerged during the course of evolution to serve these diverse needs. However, the evolutionary relationships among retinal cell types have not been mapped systematically. Here we address this gap by using single-cell transcriptomics to compare retinal cell classes, subclasses and types in 17 vertebrate species (Fig. 1b,c).

First, we show that the conserved functional and morphological character of the six cell classes is mirrored by marked cross-species similarities in gene expression. This principle extends to identified subclasses of photoreceptors, bipolar cells and amacrine cells. Transcription factors implicated in cell and subclass specification are also evolutionarily conserved, pointing to common programmes of retinal development. Within each cell class, the transcriptomic variation across species increases with evolutionary time in a manner incompatible with purely ‘neutral’ evolution^18^. Second, we assessed the extent of evolutionary variation among cell types within photoreceptors, horizontal cells, bipolar cells and RGCs, which have been comprehensively classified in mice^19–21^ and primates^22–24^. We identify numerous evolutionarily conserved types but find that variation is more extensive in RGCs than in other classes, suggesting that natural selection acts preferentially to shape the retinal output. Finally, we identify non-primate orthologues of midget RGCs, which account for more than 80% of RGCs in humans and are primarily responsible for high-acuity vision. To our knowledge, no counterparts of these cell types have previously been identified in non-primates, precluding mechanistic analysis of blinding diseases involving RGC loss, such as glaucoma. This orthology suggests that rather than appearing de novo in primates, midget RGCs evolved from cell types that were present in the common mammalian ancestor.

## Retinal cell atlases of 17 species

**Extended Data Fig. 1:**
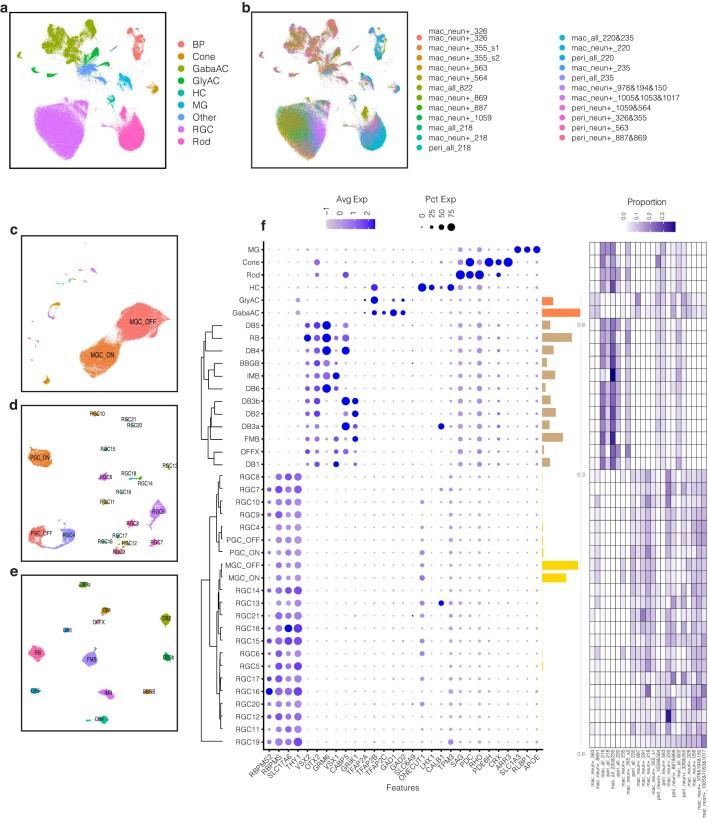
snRNA-seq data from the fovea/macula and peripheral retina of healthy human donors (n = 18). a. UMAP embedding of nuclei (n = 184,808) from the central and peripheral retina of healthy human donors, with individual points colored by cell class. PRs have been divided into rod and cone subclasses, and ACs have been divided into GABAergic and glycinergic subclasses. b. Same as **a**, with points colored by sample identity. c. UMAP embedding of RGC nuclei (n = 80,032) from the foveal and peripheral retina of healthy human donors, with individual points colored by type identity. Only ON and OFF midget ganglion RGCs are labeled. d. UMAP embedding of non-midget RGC nuclei (n = 6615) from **c**, with individual points colored by type identity. ON and OFF parasol ganglion cells are labeled. e. UMAP embedding of BC nuclei (n = 9126) from the fovea and peripheral retina of healthy human donors, with individual points colored by type identity. f. Dotplot showing expression of cell class-specific markers (columns) in the human clusters (rows). The size of each dot represents the fraction of cells in the group with non-zero expression, and the color represents expression level. The six classes are MG, HC, PR (subdivided into Rod and Cone), AC (subdivided into Gabaergic ACs (GabaAC) and glycinergic ACs (Gly AC)), BC and RGC. Only BCs and RGCs have been subclustered. Rows corresponding to BC and RGC clusters are ordered based on hierarchical clustering (dendrograms, left). Barplot on the right of the dotplot depicts the relative frequency of each cluster within a class (colors). The rightmost heatmap depicts the distribution of each cluster across biological replicates (columns).

**Extended Data Fig. 2:**
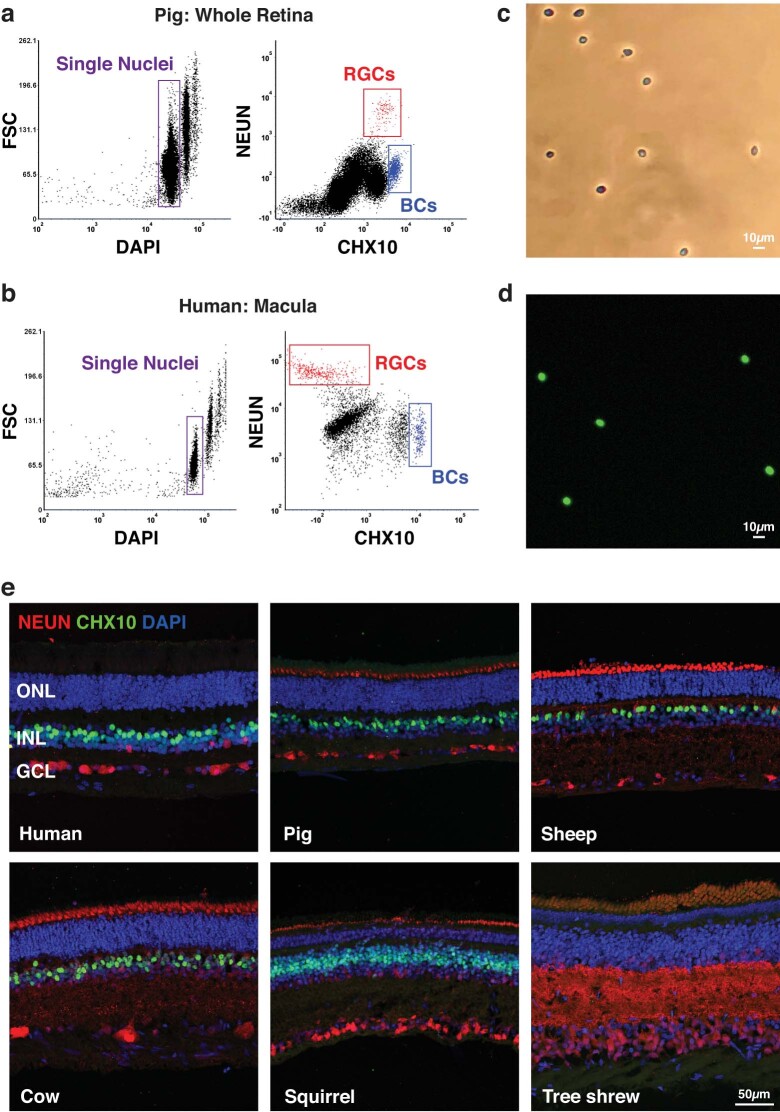
Nuclear enrichment strategies for retinal ganglion cells (RGCs) and bipolar cells (BCs). a. Examples of gating strategy in fluorescent activated cell sorting (FACS) experiments for collecting single nuclei labeled with either PE-conjugated NEUN, which enriches RGCs, or APC-conjugated CHX10 (also known as VSX2), which enriches BCs. Data shown are representative from experiments in the pig retina. NEUN and CHX10-based enrichment resulted in ~90% yield for RGCs and ~95% yield for BCs. b. Same as panel **a**, for human macular retina samples. NEUN-based enrichment resulted in ~90% yield for RGCs; BCs were not analyzed in this experiment. c. Brightfield image showing the morphology and integrity of FACS-purified nuclei. d. Confocal image of DAPI stained FACS-purified nuclei. e. Retinal sections from six species show that PE-conjugated NEUN (red) and APC-conjugated CHX10/VSX2 labels RGCs and BCs, respectively. Retinal sections were co-stained for DAPI (blue) to visualize nuclei. Scale bar, 50 μm. Images in panels **a–e** representative of n $\ge$ 3 experiments.

**Extended Data Fig. 3:**
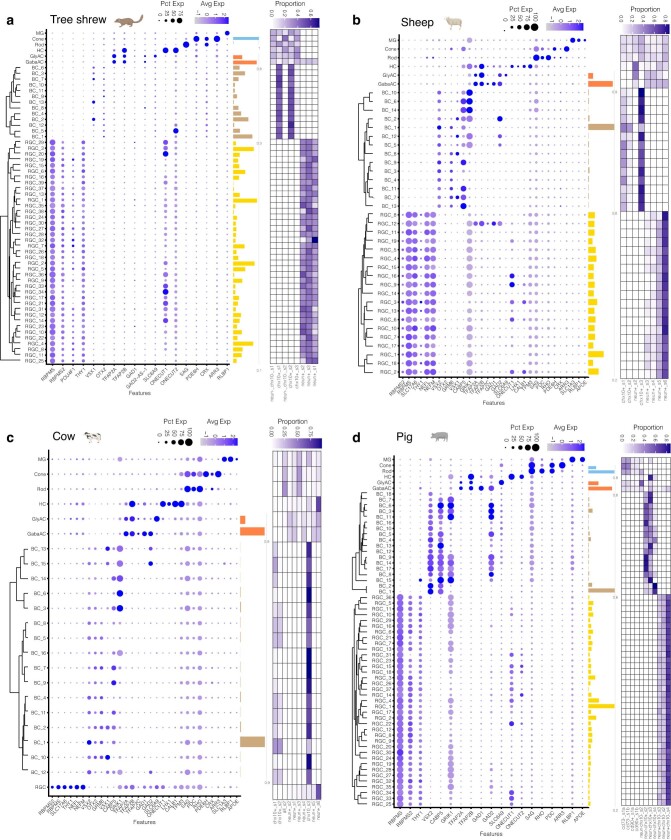
Summary of cell type atlases for tree shrew, sheep, cow, and pig. a. Dotplot showing expression of cell class-specific markers (columns) in the tree shrew (n = 3 animals; 71,571 nuclei) clusters (rows). The size of each dot represents the fraction of nuclei in the group with non-zero expression, and the color represents expression level. The six classes are MG, HC, PR (subdivided into Rod and Cone), AC (subdivided into GABAergic AC (GabaAC) and glycinergic AC (Gly AC)), BCs and RGCs. Only BCs and RGCs have been subclassified through a within-species integration and clustering analysis (**Methods**). Rows corresponding to BC and RGC clusters are ordered based on a hierarchical clustering analysis (dendrograms, left). Barplot on the right of the dotplot depicts the relative frequency of each cluster within a class (colors). The rightmost heatmap depicts the distribution of each cluster across samples (columns). Panels **b-d** depict the same information as panel **a** for sheep (n = 6 animals; 65,490 nuclei) (**b**), cow (n = 6 animals; 75,794 nuclei) (**c**), and pig (n = 4 animals; 49,955 nuclei) (**d**). Note that in this figure, as well as Extended Data Figs. 1 and 4–6, the proportions shown accurately report our data but do not necessarily represent the true endogenous proportions. This is because in many cases we depleted photoreceptors or enriched BCs or RGCs to obtain sufficient numbers of rare cell types (see Methods).

**Extended Data Fig. 4:**
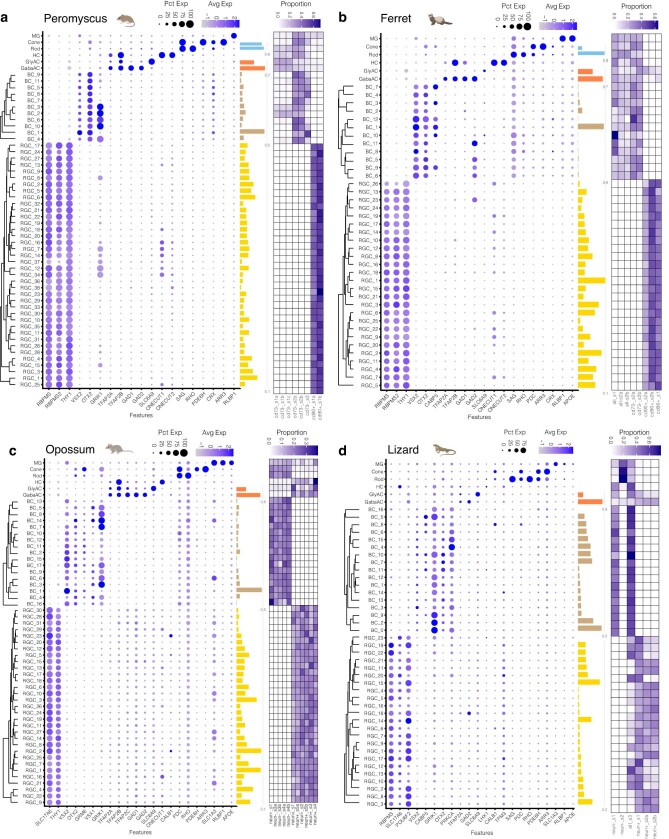
Summary of cell type atlases for Peromyscus, ferret, opossum, and brown anole lizard. Panels **a-d** depict the atlases (as in Extended Data Fig. 3) for peromyscus (n = 3 animals; 44,223 cells) (**a**), ferret (n = 2 animals; 49,972 cells) (**b**), opossum (n = 5 animals; 76,763 nuclei) (**c**), and brown anole lizard (n = 3 animals; 42,848 nuclei) (**d**).

**Extended Data Fig. 5:**
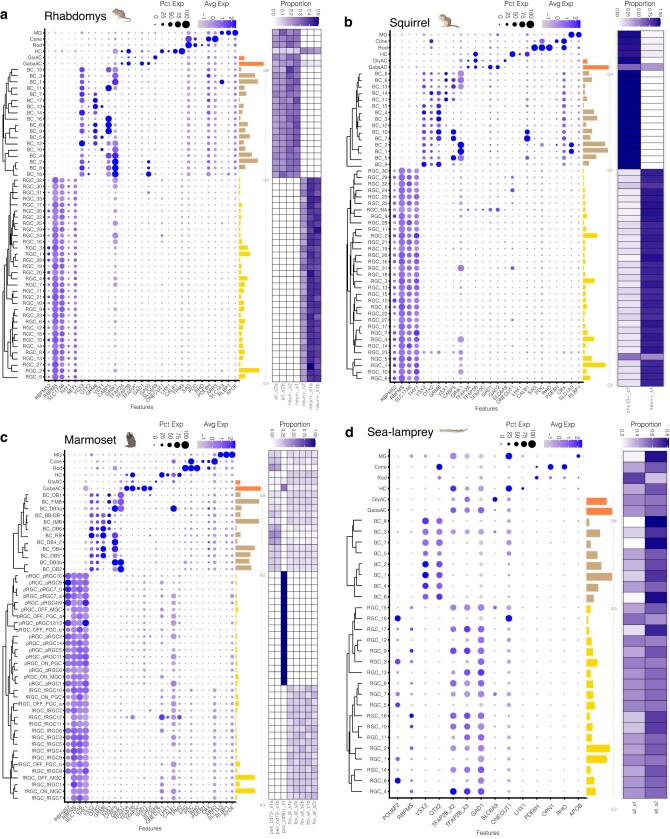
Summary of cell type atlases for Rhabdomys, squirrel, marmoset and sea-lamprey. Panels **a-d** depict atlases (as in Extended Data Fig. 3) for Rhabdomys (n = 2 animals; 65,338 nuclei) (**a**), squirrel (n = 1 animal; 22,821 cells) (**b**), marmoset (n = 2 animals; 52,559 cells) (**c**), and Sea-lamprey (n = 2 animals; 18,928 cells) (**d**).

**Extended Data Fig. 6:**
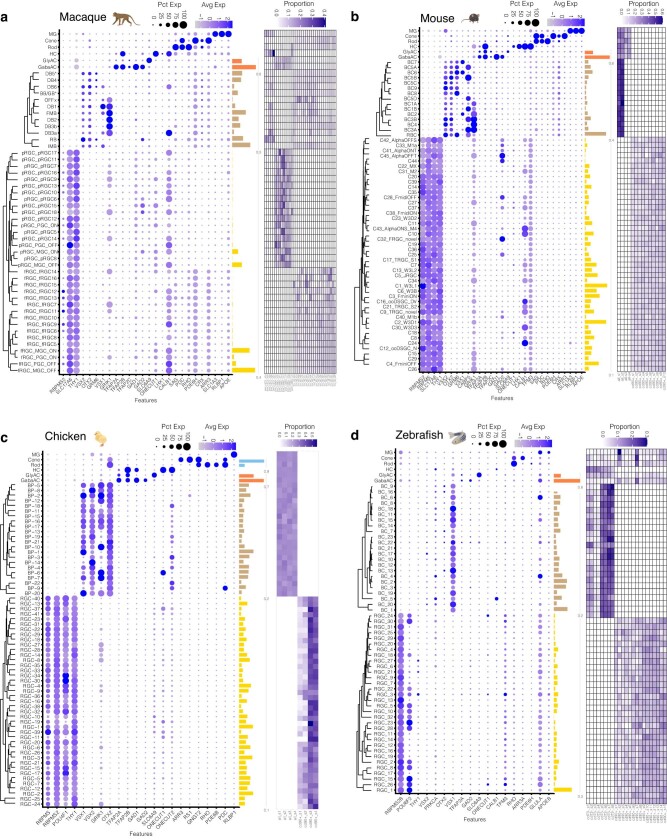
Summary of cell type atlases for macaque, mouse, chick and zebrafish. Panels **a-d** depict atlases (as in Extended Data Fig. 3) for macaque (n = 4 animals; 146,054 cells) (**a**), mouse (n = 10 animals; 51,162 cells) (**b**), chick (n = 4 animals; 34,788 cells) (**c**), and zebrafish (n = 15 biological replicates; 60657 cells) (**d**). Cluster labels are consistent with published annotations^19,20,22,27,28^. Each biological replicate in zebrafish involved a pooling of eyes from multiple (5-8) fish.

Previously, we used scRNA-seq and snRNA-seq to study retinal cell types in five species: *Mus musculus*^19,20,25,26^ (hereafter referred to as ‘mouse’), cynomolgus macaque^22^ (*Macaca fascicularis*), human^23^ (*Homo sapiens*), chick^27^ (*Gallus gallus*) and zebrafish^28^ (*Danio rerio*). For the present study, we generated atlases from 12 additional species: ferret (*Mustela putoriusfuro*), brown anole lizard (*Anolis sagrei*), deer mouse (*Peromyscus maniculatus bairdii*), tree shrew (*Tupaia belangeri chinensis*), pig (*Sus domesticus*), sheep (*Ovis aries*), cow (*Bos taurus*), opossum (*Monodelphis domestica*), marmoset (*Callithrix jacchus*), 4-striped grass mouse (*Rhabdomys pumilio*), 13-lined ground squirrel (*Ictidomys tridecemlineatus*) and sea lamprey (*Petromyzon marinus*) (Fig. 1b,c). We also profiled around 185,000 nuclei from 18 human donors, thereby allowing us to identify over 30 more cell types than had been detected in the dataset analysed previously^23^, including 10 additional RGC types (Extended Data Fig. 1). To obtain sufficient numbers of bipolar cells and RGCs for comprehensive analysis, we enriched these classes in some collections (Extended Data Figs. 2–6 and Methods). We also collected cells without enrichment to ensure representation of all classes.

We used a standardized computational pipeline to normalize, correct batch effects, reduce dimensionality and cluster the data from each species separately^29^ (Methods). Cells that did not belong to the six canonical classes named above (for example, microglia or endothelial cells) were not analysed further. Biological replicates within each collection exhibited a high degree of concordance (Extended Data Figs. 3–6). The numbers of cells in each class for each species are summarized in Supplementary Table 1.

## Molecular conservation of neuronal classes

**Fig. 2:**
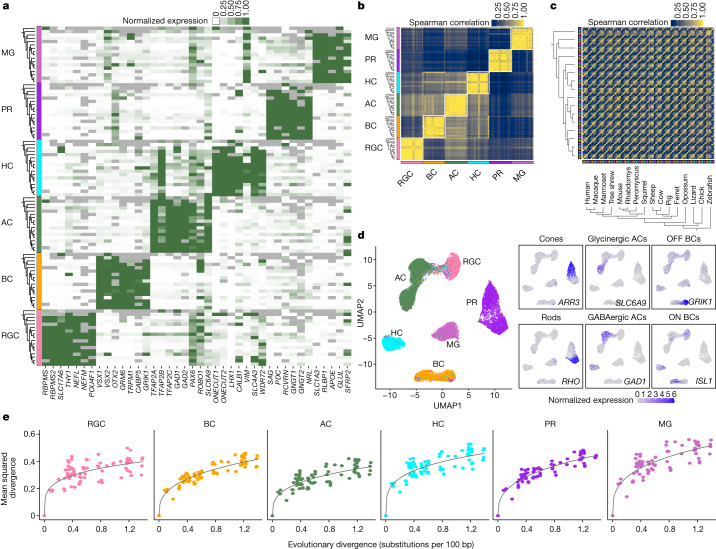
Class- and subclass-specific transcriptomic signatures. **a**, Heat map showing average expression of marker genes (columns) within each major cell class in 17 species (rows). Rows are grouped by cell class (left). Within each class, species are ordered as in Fig. 1b. Grey tiles indicate data that are missing owing to the absence of the corresponding orthologue in the species annotation. Colours indicating cell class are uniform in **a**–**e**. PR, photoreceptor. **b**, Cross-correlation matrix (Spearman) of pseudobulk transcriptomic profiles for the 16 jawed vertebrates. Rows and columns are grouped by class, and then ordered by phylogeny within a class. A total of 4,560 1:1 gene orthologues were used to calculate the correlation values. **c**, As in **b**, with rows and columns grouped by species instead of class. Matrices including lamprey are shown in Extended Data Fig. 7c,d. **d**, Left, uniform manifold approximation and projection (UMAP) embedding of integrated cross-species data, with points indicating class identity (left) or expression levels of subclass-specific markers (right). *GAD1*, a marker for GABAergic amacrine cells, is also expressed by some horizontal cells, and *ISL1*, a marker for ON bipolar cells, is also expressed by some RGCs, horizontal cells, and amacrine cells. Details of gene expression by species are shown in Extended Data Fig. 8d. **e**, Pairwise mean squared divergence of class-specific pseudobulk gene expression profiles between species (*y* axis) increases with evolutionary distance, as estimated by substitutions per 100 bp (*x* axis). Data from mammals, chicken and lizard are included. Data including zebrafish are presented in Extended Data Fig. 7e. Solid lines represent power law (*y* *=* *ax*^*b*^) regression fits. Across the graphs, $a\in [0.33,0.47]$ and $b\in [0.23,0.35]$. The coefficient of determination (*R*^2^) values range from 0.75 to 0.92. Source Data

**Extended Data Fig. 7:**
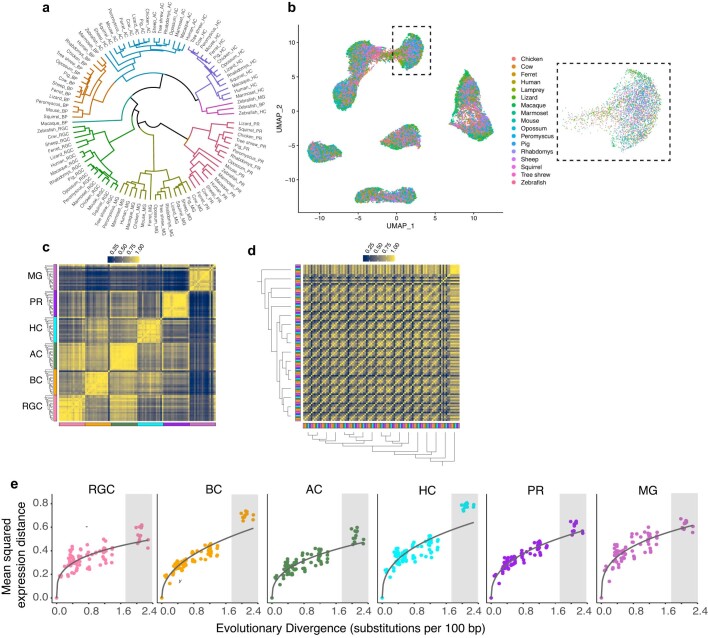
Evolutionary conservation of retinal classes. a. Dendrogram showing transcriptional relationships among pseudobulk expression vectors following integration. Each node is a cell class within a particular species. Dendrograms were computed via hierarchical clustering analysis (correlation distance, average linkage). b. Same as Fig. 2d, with cells colored by species of origin. Inset shows a magnified region containing samples from all species. c. Cross-correlation matrix (spearman) of class- and species-specific cell-averaged profiles for all 17 vertebrates (compare with Fig. 2b). Rows and columns are grouped by class, and then ordered by phylogeny within a class. d. Same as panel **c**, but rows and columns grouped based on species instead of class (compare with Fig. 2c). e. Pairwise mean-squared distance of class-specific cell-averaged gene expression profiles between all 16 jawed vertebrate species (y-axis) increases with evolutionary divergence, as estimated by substitutions per 100 bp (x-axis) (compare with Fig. 2e). Gray shaded regions demarcate species pairs involving zebrafish. Solid lines represent power law (*y = ax*^*b*^) regression fits. Across the panels, $a\in [0.34,0.47]$ and $b\in [0.29,0.45]$. The coefficient of determination (R^2^) values range from 0.79-0.93.

We analysed the expression of class markers that have been validated in mice and primates; that is, genes that are co-expressed within a retinal cell class but exhibit little or no expression in other retinal cell classes^19,20,22–26^. Many showed similar expression patterns in other vertebrates (Fig. 2a). Using these markers, we assigned cells within each species to one of the six classes. We then assessed the interspecies similarity of classes by comparing ‘pseudobulk’ transcriptomic profiles on the basis of shared orthologous genes (Methods). A cross-correlation analysis among the 16 jawed vertebrates showed that transcriptomic similarity was driven by cell class identity rather than species identity—for example, bipolar cells of a given species are more closely related to bipolar cells of other species than they are to other classes from the same species (Fig. 2b,c and Extended Data Fig. 7a,b). Qualitatively similar results were obtained when lamprey—a jawless vertebrate—was included, although the signal was attenuated because fewer orthologous genes were available (Extended Data Fig. 7c,d). Thus, class identity dominates species identity in the transcriptional profile of a retinal cell.

We found that conserved genes within a cell class included many genes encoding known lineage-determining transcription factors, such as *POU4F1* (RGCs), *VSX2* (bipolar cells and Müller glia), *OTX2* (photoreceptors and bipolar cells), *TFAP2A–C* (amacrine cells), *ONECUT1/2* (horizontal cells) and *CRX* (photoreceptors)^30^ (Fig. 2a). This suggests that the genetic mechanisms underlying neurogenesis and fate specification of cell classes are evolutionarily ancient.

We assessed evolutionary trends by comparing mean squared expression divergence in pseudobulk profiles and evolutionary distance among pairs of species for each cell class. Expression divergence increased with evolutionary distance according to a power law that was qualitatively similar across all cell classes^18^ (*R*^2^ = 0.75–0.92) (Fig. 2e and Extended Data Fig. 7e). The trends were inconsistent with purely neutral transcriptome evolution, which predicts a linear relationship between average expression distance and evolutionary distance^18,31^. Although variation at the pseudobulk level can arise from changes in cell-type composition as well as from changes in gene expression in individual cell types, the finding that the variance of Müller glia—a single cell type—was similar to that of more complex cell classes suggests that the variation at pseudobulk level is dominated by changes in gene expression in individual cell types. Thus, stabilizing and/or positive selection may contribute to the evolution of retinal cell class-specific transcriptomes.

## Molecular conservation of neuronal subclasses

**Extended Data Fig. 8:**
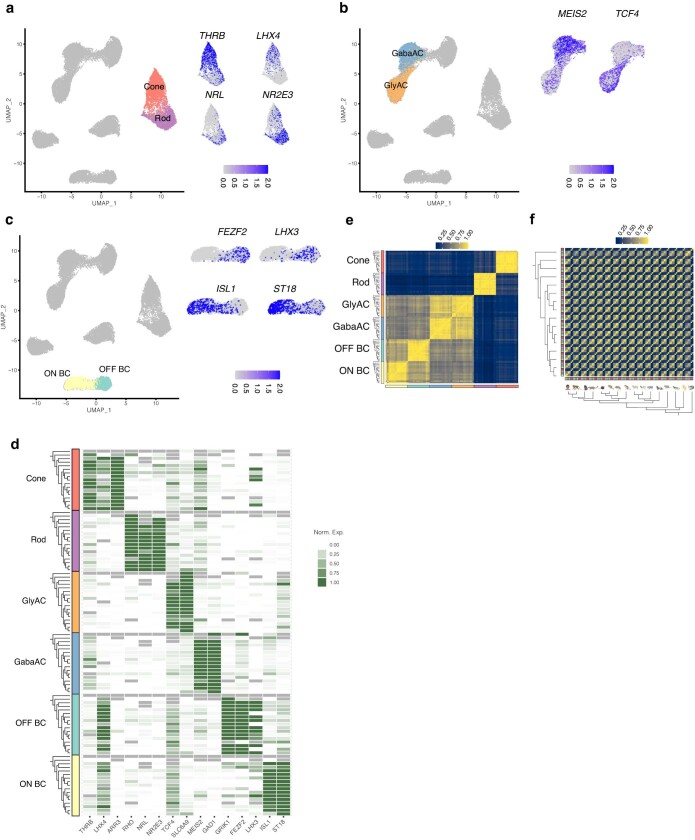
Evolutionary conservation of retinal subclasses. a. UMAP embedding of integrated cross-species data (as in Fig. 2d), highlighting PR subclasses cones and rods. Insets show feature plots of cone-specific (top) and rod-specific (bottom) transcription factors (TFs). b. Same as panel **a**, for AC subclasses GABAergic ACs (GabaAC) and glycinergic ACs (GlyAC). Insets show feature plots of a GABAergic TF *MEIS2* and a glycinergic TF *TCF4*. c. Same as panel **a**, for BC subclasses ON BCs and OFF BCs. Insets show feature plots of OFF BC-specific (top) and ON BC-specific (bottom) transcription factors (TFs). d. Heatmap showing average expression of subclass-specific genes (columns) within the six subclasses across 17 species (rows). Rows are grouped by subclass (annotation bar, left). Within each subclass, species are ordered as in Fig. 1b, with top and bottom nodes in each dendrogram corresponding to lamprey and human, respectively (corresponding to right and left in Fig. 1a). Gray tiles correspond to missing orthology information. e. Cross-correlation matrix (spearman) of subclass- and species-specific pseudobulk transcriptomic profiles for all 16 jawed vertebrates. Rows and columns are grouped by subclass, and then ordered by phylogeny within a class. Lamprey was excluded due to paucity of shared orthologs. f. Same as panel **d**, but rows and columns grouped based on species instead of subclass.

Classically, three of the retinal cell classes have been subdivided into subclasses^12^: photoreceptors comprise rods, specialized for low-light vision, and cones, which mediate chromatic vision. Nearly all amacrine cells use either GABA (γ-aminobutyric acid) or glycine as their neurotransmitter, and transmitter choice is highly correlated with key morphological features. Bipolar cells can be subdivided into those that depolarize and hyperpolarize to illumination—ON and OFF types, respectively. Within photoreceptors, amacrine cells and bipolar cells, cells from different species segregated on the basis of subclass identity and expressed orthologues of gene markers that have been well-characterized in mice (Fig. 2d and Extended Data Fig. 8). Thus, the evolutionary conservation of cell classes extends to subclasses.

Several transcription factor-encoding genes are expressed selectively in mouse retinal subclasses, including *NRL* and *NR2E3* in rods, *THRB* and *LHX4* in cones, *MEIS2* in GABAergic amacrine cells, *TCF4* in glycinergic amacrine cells, *FEZF2* and *LHX3* in OFF bipolar cells, and *ISL1* and *ST18* in ON bipolar cells^30^. Some, including *NRL*, *NR2E3*, *THRB* and *ISL1*, have been implicated in the differentiation of the subclass that expresses them. The subclass-specific expressions of these transcription factors were broadly conserved across species (Extended Data Fig. 8a–d), suggesting that the programmes specifying subclasses, like those specifying classes, are evolutionarily ancient.

## Tight conservation of outer retinal cell types

We next considered the conservation of neuronal types within classes. We began by analysing the evolutionary variation among mammalian bipolar cell types. In mice, there are 15 bipolar cell types: 6 OFF and 9 ON bipolar cell types; one of the ON bipolar cell types receives input predominantly from rods (RBCs) and all others receive input predominantly from cones^19^.

**Fig. 3:**
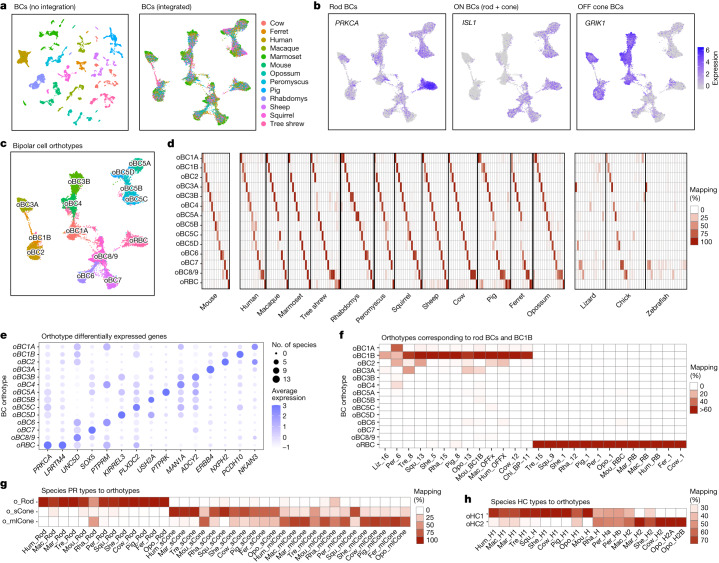
Multispecies integration of bipolar cells. **a**, UMAP of mammalian bipolar cells computed with the raw (left) and integrated (right) gene expression matrices. Cells are coloured by species of origin. **b**, Feature plots showing expression within the integrated space of the rod bipolar cell marker *PRKCA*, the ON bipolar cell marker *ISL1*, and the OFF bipolar cell marker *GRIK1*. **c**, As in **a**, but with cells coloured by orthotype identity. **d**, Left, confusion matrix showing the percentage of cells from each mouse bipolar cell-type mapping to each mammalian bipolar cell orthotype. Each column sums to 100%. See Extended Data Fig. 9a for a higher magnification view. Centre, confusion matrix showing specific mapping between mammalian bipolar cell orthotypes and bipolar cell clusters within each mammalian species (Extended Data Figs. 1 and 3–6). Right, confusion matrices showing the mapping of bipolar cell clusters in lizard, chick and zebrafish to the mammalian bipolar cell orthotype. Mapping that includes non-mammalian orthotype is shown in Extended Data Fig. 9e. **e**, Dot plot showing differentially expressed genes within each bipolar cell orthotype. The size of the dot represents the number of mammalian species (out of 13 mammalian species in total) that express the gene in at least 30% of cells in the corresponding orthotype, and the colour represents normalized expression level. **f**, Confusion matrix showing the species bipolar cell clusters (columns) that map specifically to the orthotype oRBC and oBC1B. Bipolar cell types are named on the basis of their species of origin and within-species bipolar cell cluster ID (Extended Data Figs. 1 and 3–6); for example, *Peromyscus* bipolar cell cluster 1 is called ‘Per_1’. **g**,**h**, Confusion matrix showing mapping of mammalian photoreceptor (**g**) and horizontal cell (**h**) types to orthotype. Format as in **d**, centre. Source Data

Initial clustering of mammalian bipolar cells generated groups that were defined by species (Fig. 3a). The datasets were therefore reanalysed using an integration method that minimizes species-specific signals, thereby emphasizing other transcriptomic relationships^29^ (Methods). This analysis intermixed the species while retaining structure that separates ON cone, OFF cone and ON RBCs from each other (Fig. 3b).

**Extended Data Fig. 9:**
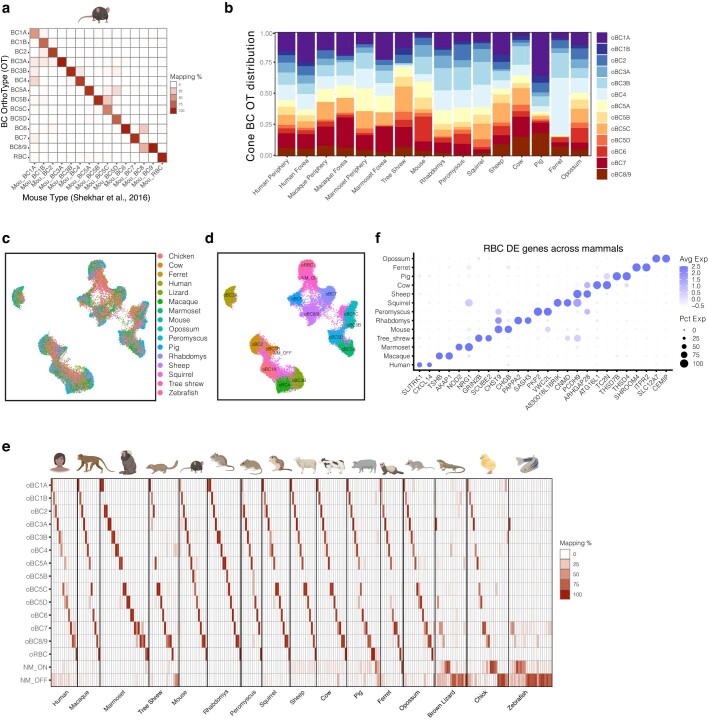
Bipolar Cell OrthoType analysis including non-mammals. a. Confusion matrix showing the rationale behind naming mammalian BC OTs (rows) based on the mapping patterns of mouse BC types (columns)^19^. Representation as in Fig. 3d, with each column summing to 100%. OT BC8/9 contains mappings from both mouse BC8 and BC9, which are transcriptionally proximal. b. Barplot showing within-species relative frequencies (y-axis) of the 13 cone BC OTs within each mammalian species (x-axis). The foveal and peripheral data from primates are plotted separately. c. Integrated UMAP of BCs from all 16 jawed vertebrates. Cells are colored by species of origin. Lamprey, a jawless vertebrate, was excluded from the analysis due to the paucity of shared orthologous genes. d. Same as **c**, with cells colored by OT identity. The integration of all jawed vertebrates recovers all the mammalian BC OTs listed in Fig. 3c, but additionally identifies two OTs enriched for non-mammalian BCs from chick, lizard and zebrafish. The two OTs, named NM_OFF and NM_ON, are enriched for OFF and ON BCs from non-mammals (also see panel **e**). e. Confusion matrices showing the mapping of species-specific BC clusters (columns) to BC OTs (rows) identified by integrating BCs from all jawed vertebrates (panel **c**). Representation as in Fig. 3d’. Mammalian BC clusters predominantly map to the mammalian OTs (rows 1-14), and the pattern of mapping is similar to Fig. 3d. Chick, Lizard and Zebrafish BCs largely map to the non-mammalian OTs NM_OFF and NM_ON (rows 15-16). f. Dotplot showing species-specific genes (columns) expressed in RBC orthologs in mammals (rows). The size and color of each dot represent the percentage of cells within the species cluster expressing the gene and the average expression level, respectively.

The integrated data revealed 14 groups of cells based on shared transcriptomic signatures (Fig. 3c). Even though species-specific cluster labels were not an input to the analysis, mouse bipolar cell types mapped to the integrated groups in a 1:1 fashion, with the sole exception of two closely related and sparsely represented types (BC8 and BC9) that mapped to the same group (Fig. 3d and Extended Data Fig. 9a). We call these groups neuronal orthotypes although, as in the case of BC8 and BC9, they may sometimes contain small sets of related types. We named the bipolar cell orthotypes according to the mouse types; thus, the orthotype containing mouse BC1A is called oBC1A, and so on. Each bipolar cell orthotype was represented in nearly all mammals (Extended Data Fig. 9b) and 91% of mammalian bipolar cell clusters (172 out of 190) predominantly mapped specifically to a single orthotype (Fig. 3d, middle and Supplementary Table 3). We identified differentially expressed genes that distinguished the bipolar cell orthotypes (Fig. 3e).

The ‘mammalian’ orthotypes remained robust when mammalian, chick, lizard and zebrafish bipolar cells were integrated together. Although 32% fewer orthologous genes were available to guide the analysis, many bipolar cell clusters in chick, several in lizard and a few in zebrafish mapped to these mammalian orthotypes (Fig. 3d, right). However, two additional ‘non-mammalian’ orthotypes emerged, comprising OFF bipolar cells and ON bipolar cells from the non-mammals (Extended Data Fig. 9c–e and Supplementary Table 3). Attempts to find additional substructure in these non-mammalian bipolar cell orthotypes were unsuccessful, probably because chick, lizard and zebrafish are nearly as evolutionarily distant from each other as they are from mammals. Nonetheless, the fact that several chick and lizard bipolar cell clusters map to the mammalian orthotypes suggests that some type-specific bipolar cell identities have been conserved for more than 300 million years.

To illustrate the utility of the integration, we highlight two bipolar cell orthotypes: oRBC and oBC1B (Fig. 3f). RBCs receive most of their input from rods, as their name implies, and they connect with specific amacrine cell types rather than connecting directly with RGCs^32^. oRBC contained RBCs from all mammals (Fig. 3f). Mammalian RBCs were distinguished by the high expression of *PRKCA* and *LRRTM4* (Fig. 3e), both of which are RBC-specific in mice^19^. RBCs also exhibit species-specific gene expression (Extended Data Fig. 9f). RBCs have been described in chicks and zebrafish, but these types did not map to oRBC.

The second orthotype represents a non-canonical OFF bipolar cell described in mice, named BC1B^19^ or GluMI^33^. The name BC1B reflects its transcriptional similarity to BC1A. However, unlike canonical bipolar cells, BC1B retracts its dendrite during early postnatal life and therefore has no direct connection with mature photoreceptors^19^. No BC1B equivalent has yet been identified in other species, probably because it lacks this connection. However, 10 of the 13 mammals profiled here, as well as chicks and lizards, contained a bipolar cell cluster that mapped exclusively to oBC1B **(**Fig. 3f), whereas two mammals (*Peromyscus* and ferret) contained a cluster that mapped to both oBC1A and oBC1B. Thus, transcriptomics enabled the identification of a potentially conserved cell type that would have been difficult to identify by conventional morphological methods; its type-specific markers can now be used to seek morphological and physiological validation.

We repeated the orthotype analysis for photoreceptors and horizontal cells, which are less diverse classes than bipolar cells. As noted above, photoreceptors are divided into two subclasses, rods and cones. Most mammals have a single rod type and two cone types, tuned to respond best to short wavelengths (S cones, also known as blue cones) and medium wavelengths (M cones, also known as green cones), respectively. However, many primates have a third cone type (L cones, also known as red cones) that is sensitive to longer wavelengths^34^. Orthotype analysis separated mammalian M and L cones from S cones effectively, with the few exceptions probably being due to insufficient cell numbers (Fig. 3g). Similarly, most mammals have two horizontal cell types, called H1 and H2—although mice and perhaps other rodents—have only a single horizontal cell type. Again, orthotype analysis separated horizontal cells into two groups (Fig. 3h). Many non-mammalian vertebrates are more complex in these respects, with 4 or 6 photoreceptor types and 4 horizontal cell types in birds (including chicken) and fish^27,34,35^ (including zebrafish); these species mapped less well onto the mammalian orthotypes.

## Retinal ganglion cell orthotypes

**Fig. 4:**
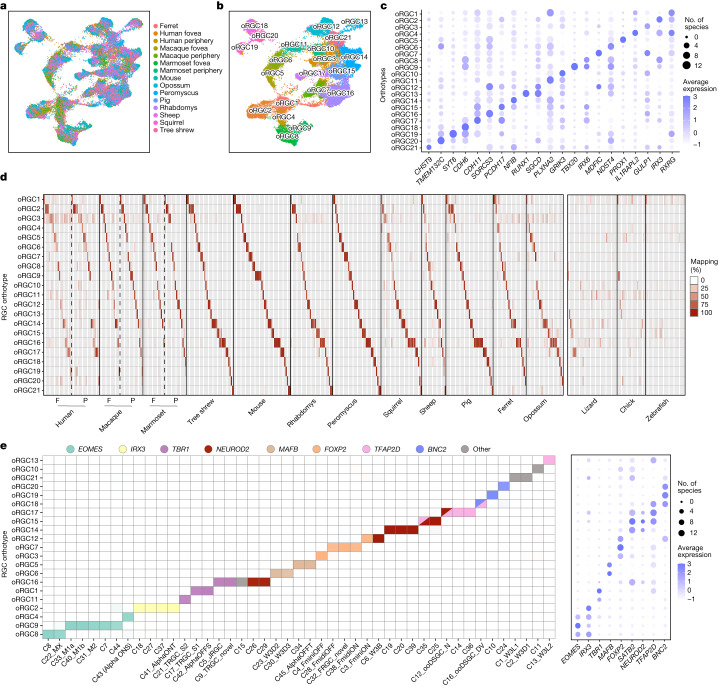
Multispecies integration of retinal ganglion cells. **a**, Integrated UMAP of RGCs from 12 mammals (cow was excluded owing to the paucity of RGC data.). Cells are labelled by species of origin. For primates, cells from fovea and periphery are plotted separately. **b**, As in **a**, with RGCs labelled by orthotype. **c**, Dot plot showing differentially expressed genes within each RGC orthotype. Representation as in Fig. 3e. **d**, Left, confusion matrices showing that species-specific RGC clusters (Extended Data Figs. 1 and 3–6) map to mammalian RGC orthotypes in a specific fashion. Representation as in Fig. 3d, centre, except that clusters from fovea (F) and periphery (P) are mapped separately for primates. Right, confusion matrices showing the mapping of RGC clusters (columns) in lizard, chick and zebrafish to the 21 mammalian RGC orthotypes. Mapping to the single non-mammalian RGC orthotype is shown in Extended Data Fig. 10d. **e**, Left, confusion matrix showing that mouse RGC types (rows; naming as in ref. ^20^) belonging to transcription factor-based subsets^39^ (colours) map to the same orthotypes (columns). Right, dot plot showing specific expression patterns of subclass-specific transcription factor-encoding genes^39^ in orthotypes. Representation as in Fig. 3e. Source Data

**Extended Data Fig. 10:**
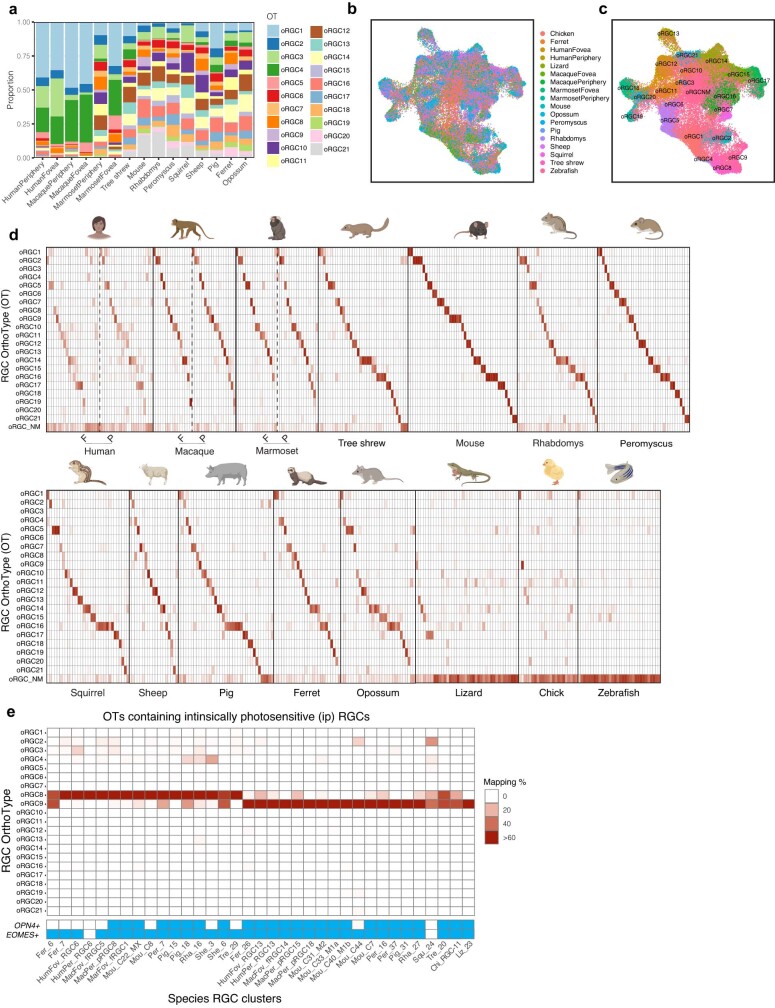
Retinal Ganglion Cell OrthoType analysis including non-mammals. a. Barplot showing within-species relative frequencies (y-axis) of the 21 RGC OTs within mammalian species (x-axis) (Fig. 4b). The foveal and peripheral data from primates are shown separately. Cow is excluded due to the paucity of data. b. Integrated UMAP of RGCs from all 15 jawed vertebrates (excluding cow). Cells are colored by species of origin. For primates, fovea and periphery are plotted separately. c. Same as **b**, with cells colored by RGC OT. OTs 1-21 map 1:1 to the mammalian OTs in Fig. 4b, but we recover an additional OT **(**NM**)** predominantly containing non-mammalian RGCs from chick, lizard and zebrafish (also see panel **d**). d. Confusion matrices showing the mapping of species RGC clusters (columns) to RGC OTs (rows) identified by integrating RGCs from all jawed vertebrates (panel **c**). Representation as in Fig. 4d. Mammalian RGC clusters predominantly map to the mammalian OTs (rows 1-21), and the pattern of mapping is similar to Fig. 4d. Except for ipRGCs, chick, lizard and zebrafish RGCs largely map to oRGC_NM (row 22). e. Confusion matrix showing the species-specific RGC clusters (columns) that map to the oRGC8 and 9, corresponding to ipRGCs. Representation as in Fig. 3f. Annotation bar (bottom) highlights species-specific RGC clusters that express *OPN4* and *EOMES*, a transcription factor expressed selectively by ipRGCs^20,21^.

We next performed orthotype analysis on RGCs, the only output neurons in the retina. We identified 21 RGC orthotypes in mammals and found differentially expressed genes that distinguished them (Fig. 4a–c and Extended Data Fig. 10a). Eighty-one per cent of mammalian RGC clusters (329 out of 408) mapped predominantly to a single orthotype (Fig. 4d). In species that contain more RGC types than orthotypes, transcriptomically similar RGC clusters mapped to the same orthotype. As was the case for bipolar cells, RGC orthotypes remained stable when lizard, chick and zebrafish were included in the integration (Fig. 4d, right), but were supplemented by an additional orthotype dominated by non-mammalian species (Extended Data Fig. 10b–d and Supplementary Table 3).

To test the reliability of orthotype analysis for RGCs, we searched for orthologues of an evolutionarily ancient set of RGC types called intrinsically photosensitive RGCs (ipRGCs). ipRGCs contain the photopigment melanopsin (encoded by *OPN4*), which enables them to generate visually evoked signals without input from photoreceptors^36^. They mediate crucial non-image-forming visual functions, such as circadian entrainment and the pupillary light reflex. ipRGCs have been detected in the retinas of diverse vertebrate orders, including several of the species profiled here, generally on the basis of *OPN4* expression^37^. ipRGCs also express the transcription factor-encoding gene *EOMES* (also known as *TBR2*), although some *EOMES*-expressing RGCs have not been functionally validated as ipRGCs. RGCs in two orthotypes, oRGC8 and oRGC9, expressed *OPN4* (Extended Data Fig. 10e). oRGC9 contained five mouse RGC types, three of which were the ipRGC types M1a, M1b and M2, which express the highest levels of melanopsin. oRGC8 contained the paralogous types, MX and C8. Overall, out of 35 clusters from 11 species in these 2 oRGCs, 25 expressed *OPN4* and 33 expressed *EOMES*. *OPN4*-expressing RGC types from chick and lizard also mapped to these orthotypes. Thus, cross-species integration captures an RGC group with a conserved physiological property.

We showed recently that 45 molecularly defined mouse RGC types, many of which map to physiologically and morphologically defined mouse RGC types^38^, can be grouped into subsets defined by selectively expressed transcription factor-encoding genes^20,39,40^. Some of these transcription factor-encoding genes (for example, *EOMES*, *TBR1* and *NEUROD2*) have been implicated in RGC development^41–44^. Although many RGC subsets defined according to transcription factor-encoding gene expression align with morphologically or functionally defined RGC subclasses (for example, *EOMES*^+^ ipRGCs and *Tbr*^*+*^ T-RGCs), others are novel (for example, *Irx3*^+^ RGCs and *Bnc2*^+^ RGCs). The mapping of mouse RGC types to RGC orthotypes mirrored these transcription factor-defined subsets (Fig. 4e, left), and subset-defining transcription factor expression patterns were recovered in a large proportion of species (Fig. 4e, right). These results suggest that as noted above for photoreceptor, bipolar cell and amacrine cell subclasses, it may be possible to classify RGCs into evolutionarily conserved subclasses.

Although orthotypes for all neuronal classes were represented in all mammals, the number of neuronal types within a species varied over a greater range for RGCs (29 ± 10 (mean ± s.d.)) than for other classes (photoreceptors, 3–4; horizontal cells, 1–2; and bipolar cells, 14 ± 2) (Extended Data Figs. 1 and 3–6). Similarly, RGC orthotypes were associated with more types within a species (1.62 ± 1.39, corresponding to a coefficient of variation (CV) of 0.86) than other classes: 1 ± 0.05, CV = 0.05 for photoreceptors; 1.1 ± 0.25, CV = 0.22 for horizontal cells; and 1.13 ± 0.44, CV = 0.4 for bipolar cells (amacrine cells are poorly annotated and cannot be integrated across species at this time). Thus, the extent of variation within cell classes increases systematically from outer to inner retina in the order photoreceptor < horizontal cell < bipolar cell < RGC.

## Orthologues of midget and parasol RGCs

In most species studied to date, no RGC type comprises more than about 10% of all RGCs. By contrast, the retina of many primates—including humans—is dominated by two closely related RGC types, ON and OFF midget RGCs, named for their diminutive dendritic trees^45^. Together they account for more than 80% of all RGCs in macaque and human, with similar abundance in fovea and periphery^22,23^. However, despite their importance for vision, no non-primate orthologues of midget RGCs have been found, and our own previous comparison of mouse and macaque primate RGCs did not find any correspondence^22^. Similarly, attempts to find orthologues of the next most abundant primate RGC types, ON and OFF parasol RGCs (5–10% of all RGCs) have remained inconclusive^2^.

**Fig. 5:**
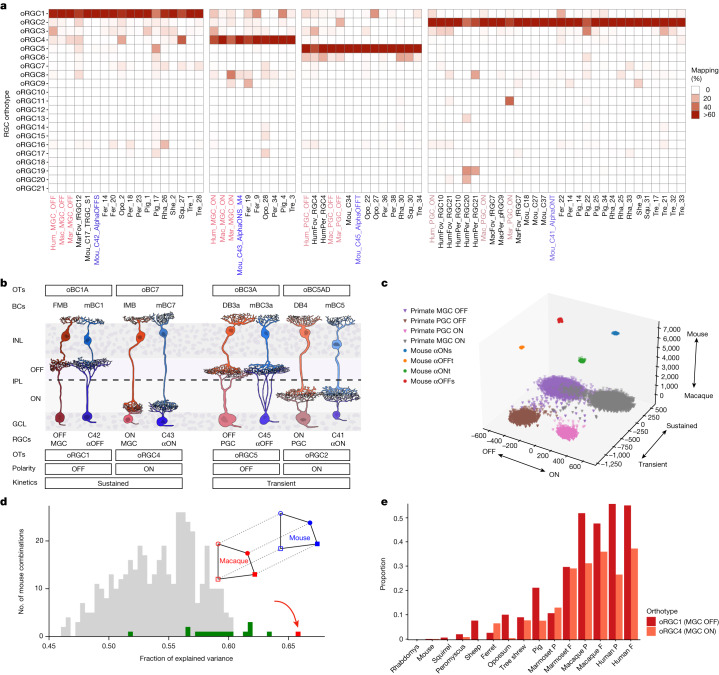
Mammalian orthologues of midget and parasol RGCs. **a**, Confusion matrix showing RGC clusters from different species that map specifically to oRGC1, oRGC4, oRGC5 and oRGC2, which contain OFF and ON midget RGCs (MGCs) and OFF and ON parasol RGCs (PGCs). Representation as in Fig. 3f. Column names corresponding to primate midget and parasol types are shown in red, and mouse α-RGC types are shown in blue. **b**, Schematic delineating morphological and physiological similarities between primate and midget RGCs and their α-RGC orthologues. Orthotypes (OTs) of each pair as well as the orthology among bipolar cell types that innervate them are also shown. Morphologies of neuronal types were created on the basis of published data (Supplementary Note 1). Within each pair, the left column corresponds to primate types and the right column corresponds to mouse types. **c**, FLDA projection of the scRNA-seq data for primate midget and parasol types and mouse α-RGC types onto the corresponding 3D space, with axes representing species, polarity and kinetics (see Supplementary Note 2). **d**, Matching MGCs and PGCs to mouse types by GAGE. Inset, given sets of mouse and primate RGC types, the model fits the arrangement of their cluster centroids in gene expression space by assuming a shape that is simply shifted to the other species via a linear translation. Symbols mark the four response types: circle, sustained; square, transient; open, ON; filled, OFF. The graph is a histogram of the fraction of explained variance showing for each proposed combination of four mouse cell types how well the resulting shape fits the macaque RGC geometry. The red bar shows the set of four α-RGC types. Green bars show combinations containing three α-RGC types. Grey bars, remaining sets of four mouse cell types as shown in Supplementary Table 4. **e**, Relative proportion of OFF and ON midget RGC orthologues in mammalian species based on frequencies of cells in oRGC1 and oRGC4. Source Data

**Extended Data Fig. 11:**
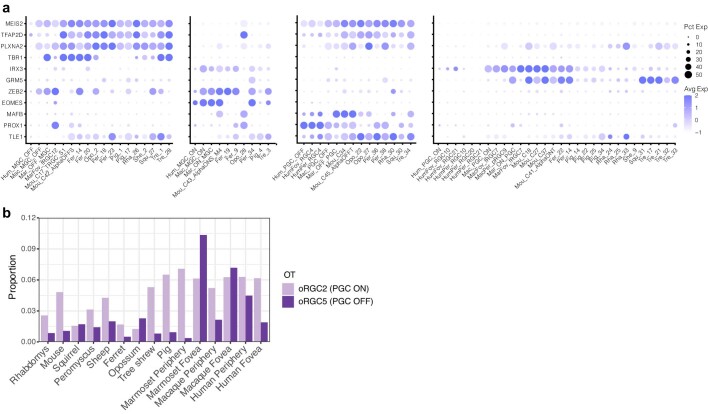
Midget and Parasol OTs. a. Dotplot showing examples of DE genes across OT1-4 and their expression across orthologous species-specific clusters. The size and color of each dot represent the percentage of cells within the species cluster expressing the gene and the average expression level, respectively. Column order as in Fig. 5a. b. Relative proportion of parasol RGC orthologs in mammalian species based on the frequencies of cells in oRGC2 and oRGC5.

We used orthotypes to revisit this issue. Each of the four abundant primate types mapped to a distinct orthotype (oRGC1, oRGC2, oRGC4 and oRGC5), and each of these orthotypes contained the corresponding cell type from both fovea and periphery of human, macaque and marmoset (Fig. 5a and Extended Data Fig. 11a). Remarkably, the mouse RGC types mapping to these orthotypes included a set of four related types called α-RGCs^46^; of the five mouse cell types mapping to the ON and OFF midget- and OFF parasol-containing orthotypes, three were α-RGCs. A resemblance of parasol RGCs to α-RGCs has been suggested previously^22,47^, but the correspondence was unexpected for midget RGCs, because α-RGCs are present at low abundance (around 2%) and are among the largest mouse RGCs. Nonetheless, several lines of evidence support the orthology between primate midgets and parasols, and the mouse α-RGC types.

First, the four α-RGC types can be distinguished on the basis of response polarity (ON versus OFF) and response kinetics (sustained (s) versus transient (t)): αONs, αOFFs, αONt and αOFFt^46^. Mouse αONs and αOFFs mapped to ON and OFF midgets, respectively, and mouse αONt and αOFFt mapped to ON and OFF parasols, respectively. Second, midgets and parasols exhibit sustained and transient light responses, respectively, that match the kinetics of their mouse orthologues^46,48^. Third, dendrites of matched types arborize in homologous sublaminae of the inner plexiform layer, with the parasol and α-transient types nearer the centre of the layer than the midget and α-sustained types^49^. Fourth, morphological studies have identified the bipolar cell types that innervate midgets, parasols and α-RGCs^50–52^. In each case, the primate bipolar cell type that provides the majority of excitatory input to the midget or parasol RGC type is a member of the same bipolar cell orthotype as a mouse bipolar cell type that provides substantial input to the corresponding α-RGC type. Thus, although none of these metadata were provided explicitly, the integration matched types correctly based on their polarity, response kinetics, dendritic lamination and inputs (Fig. 5b). In addition, orthologues exhibit similar response properties: midget RGCs and sustained α-RGCs primarily report on contrast and are minimally feature-selective, whereas parasol RGCs and transient α-RGCs, are motion-sensitive^53,54^.

**Extended Data Fig. 12:**
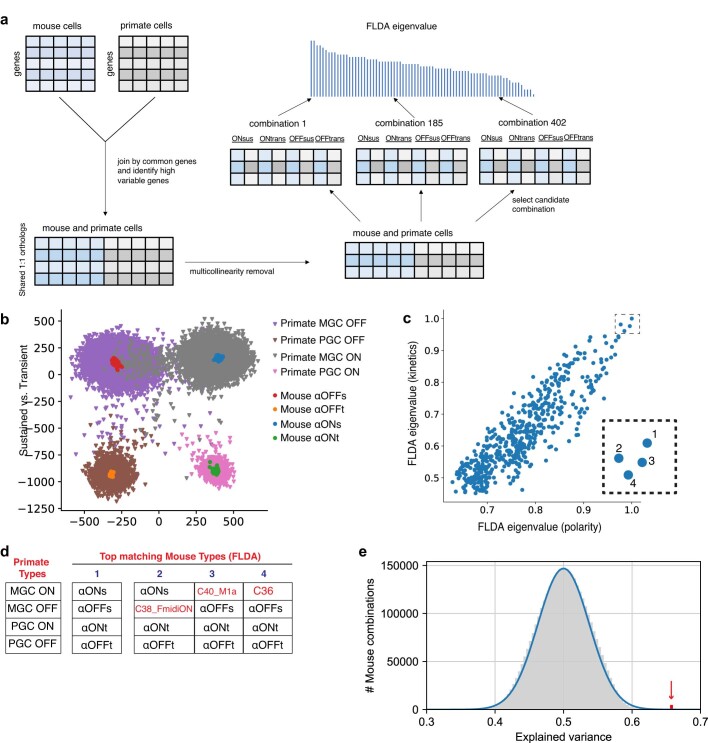
Factorized Linear Discriminant Analysis (FLDA) and Geometric Analysis of Gene Expression (GAGE). a. FLDA workflow and eigenvalue analysis. The gene expression matrices of primate and mouse RGCs were combined by their shared orthologous genes. Highly variable genes were selected, and PCA was applied to remove multicollinearity. FLDA was performed on different combinations of mouse RGC candidates with known polarity and kinetics listed Supplementary Table 4. The combinations were ranked based on their FLDA eigenvalues, which measures the variance along each attribute captured in the projection. b. Visualization of the FLDA projection (Fig. 5c) along the 2D subspace corresponding to polarity (x-axis) and kinetics (y-axis). c. Scatter plot of the FLDA eigenvalues for the kinetics (y-axis) vs. polarity (x-axis), measuring the magnitude of the variance corresponding to these attributes captured in the projection. Inset highlights the top four matches (numbered 1-4) from the 432 combinations of 4 mouse types shown in Supplementary Table 4. d. Mouse RGC types present within the top four combinations out of the 432 combinations in panel **c**. The top matched set contains all four α-RGC types; the next three include 3 α-RGC types. e. Geometric analysis of gene expression (GAGE) in which primate MGCs and PGCs are compared to all combinations of 4 mouse RGC types (45 choose 4 * 4! = 3,575,880) rather than only the 432 curated combinations used to generate Fig. 5d. Grey bars: histogram of scores for all sets of 4 mouse types. Red bar highlights the set of 4 α-RGC types with the correct matching of polarity and kinetics with the primate types, also marked by the red arrow located at a score of x = 0.657. The bulk of the distribution is approximated as a Gaussian with mean 0.50 and standard deviation 0.0374 (blue line). The 4 α-RGC fit has the second highest score among ~3.6 million candidates. The null hypothesis that this arises by chance has a p-value of p < 10^−6^ based on a one-sided Student’s t-test. The top scoring combination with a score of 0.658 involves mouse RGC types C18, C7, C39 and C8 corresponding to the ON PGC, ON MGC, OFF PGC and OFF MGC respectively. Of the four mouse types, two – C18 and C8 - have been physiologically characterized to exhibit sustained ON responses^38^, which violates their expected phenotypic correspondence to ON PGC (ON transient) and OFF MGC (OFF sustained).

We assessed the strength of the primate midget and parasol to mouse α-RGC correspondence with two additional statistical approaches. The first is factorized linear discriminant analysis^55^ (FLDA) (Extended Data Fig. 12a and Supplementary Note 2). Given single-cell transcriptomic data from cells that carry multiple categorical attributes, FLDA attempts to factorize the gene expression data into a low-dimensional representation in which each axis captures the variation along one attribute while minimally co-varying with other attributes. We applied FLDA to project primate midgets and parasols and mouse α-RGCs onto a 3D space whose three axes represent species (mouse–primate), kinetics (sustained–transient) and polarity (ON–OFF). FLDA generated a projection in which the relative arrangement of the four primate and the four mouse cell types was consistent with their attributes (Fig. 5c and Extended Data Fig. 12b). We then tested whether α-RGCs were a better transcriptomic match to midgets and parasols than other mouse RGC types carrying similar attributes. For this purpose, we identified a set of 20 mouse RGC types for which polarity (ON–OFF) and kinetics (sustained–transient) are known (Supplementary Table 4). We matched all possible 432 combinations of 4 drawn from this set with the midgets and parasols, calculated the FLDA projections, and ranked them on the basis of the magnitude of the variance captured by FLDA along the polarity and kinetics axes (Extended Data Fig. 12c). The best match comprised all four α-RGC types, and the next three matches contained three α-RGC types plus one other type (Extended Data Fig. 12d).

The second statistical method, geometric analysis of gene expression (GAGE), focuses on the geometric arrangement of the cluster means of RGC types in gene expression space (Supplementary Note 3). The cluster centroids for the macaque midget and parasol types form a four-cornered shape in the space of gene expression values. GAGE tests whether there are groups of mouse RGC types that form that same shape, except for a linear translation corresponding to species differences (Fig. 5d, inset). For every combination of four mouse cell types in the set described above, we scored how well the mouse shape matches the macaque shape (Methods). The four α-RGC types produced the strongest match by a large margin, followed by several combinations containing three α-RGC types (Fig. 5d). Finally, we considered matches for all 3,575,880 possible combinations of 4 drawn from the 45 transcriptomically defined mouse RGC types^20^. The four α-RGC types with the correct matching of polarity and kinetics with the MGCs and PGCs scored in second place out of all such combinations. The top match was biologically implausible (see Extended Data Fig. 12e).

Together, these results provide strong support for the orthology of primate midget and parasol RGCs with mouse α-RGCs, suggesting that midget and parasol RGCs are not primate innovations as they have been considered to be. Moreover, the presence of midget and parasol orthologues in all the mammals studied here (Fig. 5e and Extended Data Fig. 11b) suggests that they are likely to have evolved from antecedent types present in the mammalian common ancestor.

For midget RGCs, we suggest a relationship between their marked expansion in the primate lineage (Fig. 5e) and the evolution of visual processing. In primates, the principal retinorecipient region is the dorsolateral geniculate nucleus (dLGN), whereas in mice it is the superior colliculus^56^. Midget RGCs project almost exclusively to the dLGN^57^. In mice, anterograde^16^ and retrograde^58,59^ tracing studies suggest that α-RGCs are overrepresented among those RGCs that project to the dLGN (two- to fourfold in ref. ^53^). The dLGN provides the dominant visual input to the primary visual cortex, whereas superior colliculus projects in large part to areas that control reflexive motor responses, including eye movements^60^. In primates, complex visual processing occurs largely at the cortical level, and may be best served by the relatively unprocessed, high-acuity rendering of the visual world that midget RGCs provide. The modest loss in response time in this system is presumably compensated by the greater flexibility in response type. As the cortex has a key role in primate vision, midget-like RGCs already present in the mammalian ancestor may have decreased in receptive field size and increased in number to facilitate this flexibility as primates evolved.

## Conclusions

We integrated single-cell transcriptomic cell atlases of the retina from 17 vertebrate species and used them to assess the extent to which cell classes, subclasses and types have been conserved through vertebrate evolution. Our main results and the conclusions we draw from them are as follows. First, retinal cell classes and subclasses are highly conserved at the molecular level through evolution, mirroring their structural and functional conservation. The pattern of gene expression variation in classes is inconsistent with neutral transcriptome evolution, suggesting that selective pressures shape the cellular repertoire of the retina. Second, although greater cross-species variation exists at the level of cell types, numerous conserved types can be detected using an analytical framework that identifies transcriptomic groups, which we call orthotypes. Third, evolutionary divergence among types is more pronounced for RGCs than for other retinal classes, suggesting that the outer retina is built from a conserved parts list, whereas natural selection acts more strongly on diversifying those neuronal types that transmit information from the retina to the rest of the brain. Fourth, conserved transcription factors at all three levels (class, subclass and type) suggest that developmental programmes for the specification of retinal neurons have an ancient origin. Fifth, midget and parasol RGCs, which together comprise more than 90% of human RGCs, have orthologues in other mammalian species, suggesting that these primate cell types are derived from the expansion and modification of types present more than 300 million years ago in the retina of the last common ancestor of mammals. In mice, the orthologues are a numerically minor set of types called α-RGCs. The marked (approximately 40-fold) difference in abundance of midget orthologues between mice and humans correlates with the greater prominence of visual processing in the primate cortex. Knowing the orthologues of midget and parasol RGCs in several accessible models will aid efforts to slow their degeneration in blinding diseases such as glaucoma.

## Methods

### Ethical compliance

Human eyes were obtained post-mortem at a median of 6 h from death either from Massachusetts General Hospital via the Rapid Autopsy Program or from The Lion’s Eye Bank in Murray, UT. Acquisition and use of post-mortem human tissue samples were approved by either the Institutional Review Board of the University of Utah (protocol IRB_00010201), or the Human Study Subject Committees at Harvard (Dana Farber/Harvard Cancer Center protocol no. 13-416), and procedures were compliant with the National Human Genome Research Institute policies. All donors were confirmed to have no history or clinical evidence of ocular disease or intraocular surgery. Informed consent was obtained from all donors per IRB protocols. Pig, cow and sheep eyes were obtained, on average, 1 h after death from an abattoir located in West Groton, MA. Other animal eyes were obtained from animal colonies maintained at Brandeis University (ferret), California Institute of Technology (tree shrew), Harvard University (*Peromyscus*), MIT (marmoset), NIH (squirrel), University of Manchester, UK (*Rhabdomys*), University of Georgia (lizard) and University of California, Los Angeles (lamprey and opossum). Animals of both sexes were included when possible. Animal experiments conducted in the USA were approved by the Institutional Animal Care and Use Committees (IACUCs) in each location. *Rhabdomys* tissue was collected in accordance with the Animals, Scientific Procedures Act of 1986 (UK) and approved by the University of Manchester ethical review committee.

### Number of animals and cells or nuclei used

The number of animals used, biological replicates sequenced, and high-quality cells or nuclei collected are indicated for each species in Extended Data Figs. 1 and 3–6. The number of cells or nuclei recovered for each class within each species is indicated in Supplementary Table 1. See also ‘Statistics and reproducibility’.

### snRNA-seq

#### Nuclei isolation and sorting

For isolation of nuclei, frozen retinal tissues were homogenized in a Dounce homogenizer in 1 ml lysis buffer consisting of 0.1% NP-40 in a solution containing 10 mM Tris, 1 mM CaCl_2_, 8 mM MgCl_2_, 15 mM NaCl, 0.1 U μl^−1^ RNAse inhibitor (Promega RNasin Ribonuclease Inhibitor N2615), and 0.02 U μl^−1^ DNAse (D4527, Sigma Aldrich). The homogenized tissue was passed through a 40-µm cell strainer. The filtered nuclei were pelleted at 500 rcf for 5 min, resuspended in staining buffer (Tween 0.02% and 2% BSA in the Tris base buffer) and stained with anti-NEUN (1:300, Sigma FCMAB317PE or MAB377A5) and anti-CHX10 (1:600, Santa Cruz Biotechnology sc-365519 AF647) for 12 min at 4 °C.

Following staining, nuclei were centrifuged, resuspended in sorting buffer (2% BSA in the Tris base buffer), and counterstained with DAPI (1:1,000). The NEUN^+^ and CHX10^+^ nuclei were sorted into separate tubes using BD FACSDiva v8.02 (Extended Data Fig. 2a–c), pelleted again at 500 rcf for 5 min, resuspended in 0.04% non-acetylated BSA/PBS solution, and adjusted to a concentration of 1,000 nuclei per µl. The integrity of the nuclear membrane and presence of non-nuclear material were assessed under a bright-field microscope (Extended Data Fig. 2d,e) before loading into a 10X Chromium Single Cell Chip (10X Genomics) with a targeted recovery of 8,000 nuclei per channel.

#### Library preparation

Single-nuclei libraries were generated with either Chromium 3′ V3, or V3.1 platform (10X Genomics) following the manufacturer’s protocol. In brief, single nuclei were partitioned into Gel-beads-in-Emulsion where nuclear lysis and barcoded reverse transcription of RNA would take place to yield cDNA; this was followed by amplification, enzymatic fragmentation and 5′ adapter and sample index attachment to yield the final libraries. Libraries were sequenced on an Illumina NovaSeq at the Bauer Core Facility at Harvard University. Sequencing data were demultiplexed and aligned using Cell Ranger software (version 4.0.0, 10X Genomics).

### Histology

Whole eyes were fixed in 4% paraformaldehyde (in PBS) for 1–2 h and then transferred to PBS. Either whole retinas or 8-mm punches of central retina were dissected out and sunk in 30% sucrose in PBS overnight at 4 °C, before being embedded in tissue freezing medium and sectioned coronally at 20 μm in a cryostat. Sections were mounted onto coated slides. Slides were incubated for 1 h with 5% donkey serum (with 0.1% Triton X-100) at room temperature, then overnight with primary antibodies (1:500 RBPMS (PhosphoSolutions 1832-RBPMS); 1:400 CHX10 (Novus Biologicals NBP1-84476); 1:50 AP2A (DSHB 3B5)) at 4 °C, and finally for 2 h with secondary antibodies in PBS at room temperature. Images were acquired on Zeiss LSM 900 confocal microscopes with 405, 488, 568 and 647 nm lasers, and processed using Zeiss ZEN software suites.

### Preprocessing of transcriptomic data

We used Cellranger (v7.0, 10X Genomics) to align the scRNA-seq and snRNA-seq datasets, following the manufacturer’s instructions. For each species, sequencing reads were demultiplexed into distinct samples and the.fastq.gz files corresponding to each sample were aligned to reference transcriptomes to obtain binary alignment map (.bam) files. The reference transcriptomes used are listed in Supplementary Table 5. To include both exonic and intronic reads in the quantification of gene expression for each sample, regardless of cellular or nuclear origin, we applied velocyto^61^ to the corresponding.bam files. This generated two separate gene expression matrices (GEMs) (genes × cells) for each sample, corresponding to ‘spliced’ and ‘unspliced’ reads. The two GEMs were summed element by element to obtain the ‘total’ GEM for each sample. For each species, GEMs from different samples were combined (column-wise concatenated) to yield a species GEM.

### Computational analysis

Analysis of the GEMs was performed in R. Our workflow was based on Seurat v4.3.0 for single-cell analysis developed and maintained by the Satija laboratory^29,62^ (https://satijalab.org/seurat/) and includes several packages used for statistical calculations and data visualizations including MASS v7.3.60, pvclust v2.2.0, reshape2 v1.4.4, stats v4.3.0, ggplot2 v3.4.2, dendextend v1.17.1 and ggdendro v0.1.23 We describe the analysis steps here at a high level. We have also made the analysis scripts available via Zenodo (https://zenodo.org/record/8067826) and on our Github page (https://github.com/shekharlab/RetinaEvolution).

#### Segregation of major retinal cell classes

Data from each species were separately analysed through a clustering procedure to identify high-quality cells, and segregate the major cell classes (photoreceptor, bipolar cell, horizontal cell, amacrine cell, RGC and Müller glia). In brief, GEMs from different replicates were combined, and transcript counts in each cell was normalized to a total library size of 10,000 and log-transformed (*X* → log (*X* + 1)). We identified the top 2,000 highly variable genes and applied principal components analysis to factorize the submatrix corresponding to these highly variable genes. Using the subspace corresponding to the top 20 principal components, we built a *k*-nearest neighbour graph on the data, and then clustered with a resolution parameter of 0.5 using Seurat’s FindClusters function. The same principal components were used to embed the cells onto a 2D visualization using the uniform manifold approximation^63^. The 2D embeddings were solely used to visualize clustering structure and gene expression patterns post hoc.

Each cluster was assigned to one of the six major retinal cell classes based on expression of orthologues of canonical markers characterized in mice^25^: photoreceptors (*Arr3*, *Rho* and *Crx*), horizontal cells (*Calb1*, *Onecut1*, *Onecut2* and *Lhx1*), bipolar cells (*Vsx1*, *Otx2* and *Grik1*), amacrine cells (*Gad1*, *Gad2*, *Tfap2a*, *Tfap2b* and *Tfap2c*), RGCs (*Rbpms*, *Nefl*, *Nefm* and *Slc17a6*) and Müller glia (*Glul*, *Apoe* and *Rlpb1*). Clusters that mapped to other cell types found at much lower frequency (such as endothelial cells or microglia) or that contained low quality cells were not considered further. The number of cells of each class in each species is indicated in Supplementary Table 1. We note that because many experiments were designed to enrich certain classes (RGCs or bipolar cells), the relative frequencies do not reflect endogenous values.

#### Integration and clustering to identify species-specific types for photoreceptors, horizontal cells, bipolar cells and RGCs

We separated photoreceptors, horizontal cells, bipolar cells and RGCs within each species, and clustered them independently using the following procedure. After subsetting the data by class, cells with abnormally high (>mean + 2 × s.d.) or low (<mean − 2 × s.d.) counts were removed. We also removed replicate batches that contained the class of interest at a frequency less than 50 cells. We split the cells by replicate ID and used Seurat’s integration pipeline to remove batch effects, reduce dimensionality and cluster the data in a shared low-dimensional integrated space. We selected the top 20–25 latent variables in the integrated space to identify clusters and generate 2D UMAP visualizations.

We initially deliberately overclustered the data using a resolution parameter of 1.1. Clusters were then merged or pruned as follows: for each cluster, we calculated differentially expressed marker genes, and these markers were inspected to determine if clusters should be merged or removed. Some clusters were also removed if their top differentially expressed markers were widely expressed in several clusters, if they had lower RNA counts compared to other clusters, or if several of the top differentially expressed markers were canonical markers for contaminant cell classes. If more than 20% of cells were removed via pruning, the filtered data was subjected to another round of integration and clustering. Two or more clusters were merged if a differential expression test failed to find markers that sufficiently distinguished the clusters.

We applied these steps to define photoreceptor, horizontal cell, bipolar cell and RGC clusters for species initially reported in this paper: *Peromyscus*, ferret, opossum, brown anole lizard, cow, sheep, pig, 13-lined ground squirrel, 4-striped grass mouse, marmoset and tree shrew. Individual clusters correspond to individual cell types, and in some cases, to small groups of closely related types. For the sake of consistency, we also applied the same procedure to photoreceptor, horizontal cell, bipolar cell and RGC data of species published elsewhere (mouse^19,20^, macaque^22^, human^23^, zebrafish^28^ and chick^27^). In all cases, our clusters were largely consistent with published annotations, and we therefore labelled these clusters based on their published labels.

#### Selection of shared orthologous genes

Orthologous genes were identified using orthology tables via Ensembl BioMart (https://useast.ensembl.org/info/data/biomart/index.html). Using mouse as a reference species, pairwise orthology tables were generated between mouse and every other species. These orthology tables contained information about the number of predicted orthologues for every mouse gene within each species. Mouse genes that had a 1:1 orthologue in every other species were retained as the set of orthologous features, with the exception of zebrafish. Due to a whole gene duplication, zebrafish has several paralogous pairs of genes (for example, *rbpms2a* and *rbpms2b*) known as ‘ohnologs’^64^. The prevalence of ohnologs results in a paucity of 1:1 orthologues. To address this issue, we collapsed each ohonolog pair by summing over their expression (for example, *rbpms2a* and *rbpms2b* to *rbpms2*). If the ohnologs were the only orthologues of a gene, then the composite gene was regarded as the 1:1 orthologue for further analysis. Overall, we found 1,905 1:1 orthologues among all 17 species, 4,560 among the 16 jawed vertebrates (that is, omitting lamprey) and 6,693 among the 13 mammals. The number of shared orthologues decreased with evolutionary distance, and we found fewer orthologues shared between mammals and non-mammalian vertebrates than among mammals.

#### Visualization of cell classes

For an alternative view on the cell classes, we subsampled each cell class to 200 per species, and then combined the GEMs. The resulting GEMs were integrated using Seurat using each species as a ‘batch’. Note that batch correction was not performed for samples within a species, nor was cell class information provided to the integration. The resulting integrated data was visualized on a UMAP (Fig. 2d and Extended Data Fig. 8). Dendrograms for the cell-averaged profiles were constructed using hclust (package stats), and then plotted in a circular representation using the circlize_dendrogram function (package dendextend) (Extended Data Fig. 7a).

#### Evolutionary variation of pseudobulk transcriptomes

For each species, we computed cell-averaged (or pseudobulk) gene expression vectors for the six major cell classes (photoreceptor, horizontal cell, bipolar cell, amacrine cell, RGC and Müller glia). Each pseudobulk vector was *z*-scored (subtract mean and divide by variance) prior to subsequent computations. The mean squared expression distance (MSD) between two species for a cell class was calculated as the euclidean distance between the corresponding pseudobulk vectors ${\rm{MSD}}\left(a,b\right)={\left|\left|a-b\right|\right|}^{2}$. To analyse evolutionary trends within a class (Fig. 2e), we compared ${\rm{MSD}}\left(a,b\right)$ to evolutionary time separating the corresponding species $t\left(a,b\right)$. To estimate the evolutionary time for each pair of species, we downloaded a phylogenetic tree of vertebrate species from the UCSC Genome Browser at http://hgdownload.cse.ucsc.edu/goldenpath/hg19/multiz100way/^65^. Evolutionary time separating two pairs of species was assumed to be the branch length between the corresponding nodes of this tree, measured in units of substitutions per 100 bp of neutrally evolving sites. Branch lengths were computed using the Environment for Tree Exploration toolkit^66^. We then fit the MSD versus *t* using a power law model, ${\rm{MSD}}=a{t}^{b}$ introduced earlier^18^, which is reported in Fig. 2e and Extended Data Fig. 7e. We also attempted to fit the data with a linear model ${\rm{MSD}}=a+bt$ and an Ornstein–Uhlenbeck model ${\rm{MSD}}=a(1-{e}^{-bt})$ but both produced fits with lower *R*^2^ than the power law model.

#### Data integration and identification of orthotypes

We identified orthotypes separately for photoreceptors, horizontal cells, bipolar cells and RGCs. In each case, we followed the following steps: (1) Within each species, the corresponding GEM for each type was downsampled cluster-wise to include no more than 200 cells per cluster. This ensures equitable representation of the transcriptomic clusters indicated in Extended Data Figs. 3–6; (2) the downsampled species-specific GEMs were combined along the set of shared gene orthologues, normalized to 10,000 counts per cell, and log-transformed; (3) 2,000 highly variable genes were selected within each species, and features that were repeatedly variable were used for anchor finding, integrated dimensionality reduction, and clustering of GEMs based on the Seurat pipeline^29^. The resulting clusters were called orthotypes. A resolution of 0.5 was used for the clustering. Transcriptomically proximal orthotypes based on a gene expression dendrogram that contained distinct subsets of species were merged. Note that other than the downsampling step, species cluster IDs were not used to influence the selection of variable genes, integration or clustering steps.

#### Integrating mammalian and non-mammalian datasets

In several cases, cells from non-mammalian species formed orthotypes separate from those containing cells from mammalian species. We believe that this result largely reflects three issues. First, the representation of species classes in our study is skewed: 13 mammals vs 1 reptile, 1 bird and 1 fish. Second, non-mammalian species are generally more evolutionarily distant from each other than mammalian species are from each other. Third, the number of 1:1 orthologous genes decreases as more distant species are co-analysed, which further compromises integration due to the loss of features. Including additional non-mammalian species and or improving computational methods may lead to greater inclusion of non-mammalian cell types in the current mammalian orthotypes.

### Statistics and reproducibility

Based on the cluster-informed downsampling procedure described above, *n* = 32,350 cells of multiple cell classes were used to generate Fig. 2d, and 38,366 bipolar cells, 61,161 RGCs, 13,605 photoreceptors and 5,405 horizontal cells were used to generate the orthotype results shown in Figs. 3 and 4. The mammalian orthotypes remained robust to different downsampling trials (see below), as well as the inclusion of non-mammals in the analysis (refer to Fig. 3d and Extended Data Fig. 9d for bipolar cells, and Fig. 4d and Extended Data Fig. 10c for RGCs). Across downsampling trials, we found that cells mapping to a given orthotype were present in the same cluster >90% of the time. As the orthotypes are the result of a clustering of the integrated data, the number of orthotypes depends on the resolution parameter. We varied the clustering resolution and tracked the number of orthotypes, the adjusted Rand index (ARI) of the clustering, and the number of species-specific orthotypes. The bipolar cell orthotypes were robust across a wide range of resolution (0.4–1.5), as indicated by a stable number of orthotypes (16–21), high values of the ARI (0.88–0.96), and very few, if any, species-specific orthotypes. The RGC orthotypes exhibited higher sensitivity to the resolution parameter over the same range, with the number of clusters ranging from 26–46. For resolution values over 1, moret than 5 species-specific orthotypes were consistently observed across trials. However, ARI values were reasonably high across values tested (0.625–0.849). The results presented in the main text are for a resolution of 0.5.

We repeated the orthotype analysis for bipolar cells using three alternative integration methods: Harmony^67^, Liger^68^ and scVI^69^. All three methods produced results consistent with those from Seurat, but they generated several additional species-specific orthotypes and also did not resolve some known distinctions among bipolar cell types. We therefore used Seurat to obtain the results presented in the text.

### Factorized linear discriminant analysis

FLDA seeks a low-dimensional factorization of high-dimensional gene expression data from cells with multiple categorical attributes such that each axis of the low-dimensional space captures the variation along one attribute while minimizing co-variation with other attributes. The mathematical derivations underlying FLDA are described in a previous paper^55^, and are summarized in Supplementary Note 2. In this study, we applied FLDA to factorize transcriptomic data for RGCs carrying three categorical attributes: response polarity (ON vs OFF), response kinetics (transient vs sustained) and species (mouse vs primate). Using A, B and C to represent these attributes, the total gene expression covariance matrix can be expressed as:

$${\varSigma }_{{\rm{T}}}={\varSigma }_{{\rm{A}}}+{\varSigma }_{{\rm{B}}}+{\varSigma }_{{\rm{C}}}+{\varSigma }_{{\rm{e}}}$$

where ${\varSigma }_{{\rm{T}}}$ is the total covariance matrix, and ${\varSigma }_{{\rm{A}}}$, ${\varSigma }_{{\rm{B}}}$ and ${\varSigma }_{{\rm{C}}}$ are covariance explained by attributes A, B and C respectively. ${\varSigma }_{{\rm{e}}}$ is the residual variance that is not explained by these attributes.

FLDA identifies a 3D embedding (**u**, **v**, **w**) of the cells such that **u** maximizes the variance of attribute A while minimizing variances of attributes B and C, **v** maximizes the variance of attribute B while minimizing variances of attributes C and A, and **w** maximizes the variance of attribute C while minimizing variances of attributes A and B. Supplementary Note 2 shows that **u**, **v** and **w** are solutions to generalized eigenvalue problems.

### Geometric analysis of gene expression

This approach is similar in intent to FLDA in that the goal is to identify axes in gene expression space that capture the structure of the data, and that the choice of these axes is guided by a structure imposed through a Cartesian classification of cell types (for example ON vs OFF or primate vs mouse). The main difference is that FLDA also attempts to capture the variance across cells within a type, and this influences the selection of the composite axes **u**, **v** and **w**. By contrast, GAGE only seeks to model the shape formed by the gene expression centroids of the cell types under consideration. Thus, for a quartet of primate cell types (MGC OFF, MGC ON, PGC OFF and PGC ON) that form some shape in gene expression space, this method asks if there is a quartet of mouse cell types that forms the same shape. The mathematical and implementation details of this method are delineated in Supplementary Note 3.

### Reporting summary

Further information on research design is available in the Nature Portfolio Reporting Summary linked to this article.

## Online content

Any methods, additional references, Nature Portfolio reporting summaries, source data, extended data, supplementary information, acknowledgements, peer review information; details of author contributions and competing interests; and statements of data and code availability are available at 10.1038/s41586-023-06638-9.

### Supplementary information

Supplementary Information Supplementary Notes 1–3, Supplementary Table and references.

Reporting Summary

Supplementary Table 1 Numbers of cells and nuclei per retinal class per species that passed quality metrics. Note that the proportions do not represent endogenous values, but reflect the results of antibody-based enrichment, which was directed to recover BCs and RGCs in many samples.

Supplementary Table 2 Key between sample names and identifiers in Extended Data Figs. 1 and 3–6.

Supplementary Table 3 List of species-specific cell types represented in BC and RGC orthotypes.

Supplementary Table 4 List of mouse RGC types with known polarity and kinetics tested for matching with primate ON/OFF midget and ON/OFF parasol RGC types using FLDA.

Supplementary Table 5 List of reference transcriptomes used to align scRNA-seq and snRNA-seq data from species analysed in this study.

### Source data

Source Data Fig. 2

Source Data Fig. 3

Source Data Fig. 4

Source Data Fig. 5

## Data Availability

The raw and processed sequencing data produced in this work are available via the Gene Expression Omnibus (GEO) under accession GSE237215. The species-specific datasets are available via the subseries accession numbers GSE237202–GSE237214. Previously published data utilized in this paper were downloaded from GEO repositories with accession numbers GSE81905, GSE137400, GSE152842, GSE148077, GSE15910 and GSE236005. Species phylogenetic trees were downloaded from the UCSC Genome Browser database (https://genome.ucsc.edu), and species reference genomes are available on Ensembl (https://www.ensembl.org). Source data are provided with this paper.

## Code availability

scRNA-seq data clustering, integration and visualization was performed in the R statistical language, and heavily relied on the Seurat package (https://satijalab.org/seurat/). All scripts are available via Zenodo (https://zenodo.org/record/8067826) and on our GitHub page (https://github.com/shekharlab/RetinaEvolution). FLDA analysis was performed in Python, and the code and documentation are available at https://github.com/muqiao0626/FLDA. GAGE analysis was performed in Python, and the code and documentation are available at https://github.com/markusmeister/Gene-Geometry.
