# Supplementary material for: Evolution of neuronal cell classes and types in the vertebrate retina
Source: Nature. 2023 Dec 13;624(7991):415–24. doi: 10.1038/s41586-023-06638-9 (PMC10719112; doi:10.1038/s41586-023-06638-9)
Supplement: Supplementary file 1 — Supplementary Notes 1–3, Supplementary Table and references. [file 41586_2023_6638_MOESM1_ESM.pdf]

nature portfolio

https://doi.org/10.1038/s41586-023-06638-9

Supplementary information

Evolution of neuronal cell classes and types in the vertebrate retina

In the format provided by the authors and unedited

Supplementary Note 1

Approximate morphologies and stratification of four types of cone bipolar cells and four types of RGCs in mouse and primate retinas were drawn based on the illustrations from previous literature listed in the table below.

Table 1: A list of figures in previous reports that were used to draw rough morphology of bipolar cells and RGCs depicted in **Fig. 5b**.

**Class**	**Type**	**Speceis**	**Source**
BC	BC1, BC7, BC3a, BC5, FMB, IMB, DB3a, DB4	*M. musculus, M. fascicularis*	Fig. 8 in Ref. 1
BC	BC1, BC7, BC3a, BC5	*M. musculus*	Fig. 1b in Ref. 2
BC	BC1, BC7, BC3a, BC5	*M. musculus*	Fig. 2 in Ref. 3
BC	FMB, IMB, DB3a, DB4	*M. fascicularis*	Fig. 1 in Ref. 4
RGC	ON Midget, ON Parasol	*M. fascicularis, H sapiens*	Fig. 9 in Ref. 4
RGC	ON Parasol, OFF Parasol, OFF midget	*M. fascicularis, H sapiens*	Fig. 4a in Ref. 5
RGC	α-OFFt	*M. musculus*	Fig. 2a in Ref. 6
RGC	α-OFFs, α-ONs, α-OFFt	*M. musculus*	Fig. 3 in Ref. 7
RGC	α-OFFs, α-ONs, α-OFFt, α-ONt	*M. musculus*	Fig. 4b in Ref. 8

References

1 Ghosh, K. K., Bujan, S., Haverkamp, S., Feigenspan, A. & Wässle, H. Types of bipolar cells in the mouse retina. Journal of Comparative Neurology 469, 70-82 (2004).
2 Euler, T., Haverkamp, S., Schubert, T. & Baden, T. Retinal bipolar cells: elementary building blocks of vision. Nature Reviews Neuroscience 15, 507-519 (2014).

3 Tsukamoto, Y. & Omi, N. Classification of Mouse Retinal Bipolar Cells: Type-Specific Connectivity with Special Reference to Rod-Driven AII Amacrine Pathways. Frontiers in Neuroanatomy 11, doi:10.3389/fnana.2017.00092 (2017).
4 Tsukamoto, Y. & Omi, N. ON bipolar cells in macaque retina: type-specific synaptic connectivity with special reference to OFF counterparts. Frontiers in Neuroanatomy 10, 104 (2016).
5 Schiller, P. H. Parallel information processing channels created in the retina. Proceedings of the National Academy of Sciences 107, 17087-17094 (2010).
6 Ran, Y. et al. Type-specific dendritic integration in mouse retinal ganglion cells. Nature Communications 11, 2101 (2020).
7 Sanes, J. R. & Masland, R. H. The types of retinal ganglion cells: current status and implications for neuronal classification. Annual review of neuroscience 38, 221-246 (2015).
8 Krieger, B., Qiao, M., Rousso, D. L., Sanes, J. R. & Meister, M. Four alpha ganglion cell types in mouse retina: Function, structure, and molecular signatures. PloS one 12, e0180091 (2017).

Supplementary Note 2: Factorized Linear Discriminant Analysis (FLDA) to find mouse RGC types that optimally match primate midget and parasol RGC types

Mathematical details of FLDA

For a detailed description of the FLDA method, please refer to our previous work [5]. In brief, FLDA is a method that projects high-dimensional gene expression data from cells with multiple categorical attributes into a low-dimensional space where each axis captures the variation along one attribute while minimizing co-variation with other attributes.

In this study, we used FLDA to analyze three categorical attributes of retinal neurons: response polarity (ON vs. OFF), response kinetics (transient vs. sustained), and species (mouse vs. primate). Let's use A, B, and C to represent these attributes. i, j, k denote the indices of attributes A, B, and C, and a, b, c are the number of categories in attributes A, B, and C. n_{ijk} is the number of cells in the category combination ijk. $\boldsymbol{x}_{ijkl}$ is the gene expression vector of the lth cell in the category combination ijk.

The covariance matrix of total variance can be decomposed as:

$$\boldsymbol{\Sigma}_T = \boldsymbol{\Sigma}_A + \boldsymbol{\Sigma}_B + \boldsymbol{\Sigma}_C + \boldsymbol{\Sigma}_e \tag{1}$$

where

$$\boldsymbol{\Sigma}_T = \sum_{i=1}^{a}\sum_{j=1}^{b}\sum_{k=1}^{c}\left[\frac{1}{n_{ijk}}\sum_{l=1}^{n_{ijk}}(\boldsymbol{x}_{ijkl} - \boldsymbol{m}_{...})(\boldsymbol{x}_{ijkl} - \boldsymbol{m}_{...})^\top\right] \tag{2}$$

$$\boldsymbol{\Sigma}_A = bc\sum_{i=1}^{a}(\boldsymbol{m}_{i..} - \boldsymbol{m}_{...})(\boldsymbol{m}_{i..} - \boldsymbol{m}_{...})^\top \tag{3}$$

$$\boldsymbol{\Sigma}_B = ac\sum_{j=1}^{b}(\boldsymbol{m}_{.j.} - \boldsymbol{m}_{...})(\boldsymbol{m}_{.j.} - \boldsymbol{m}_{...})^\top \tag{4}$$

$$\boldsymbol{\Sigma}_C = ab\sum_{k=1}^{c}(\boldsymbol{m}_{..k} - \boldsymbol{m}_{...})(\boldsymbol{m}_{..k} - \boldsymbol{m}_{...})^\top \tag{5}$$

and

$$\boldsymbol{\Sigma}_e = \boldsymbol{\Sigma}_T - \boldsymbol{\Sigma}_A - \boldsymbol{\Sigma}_B - \boldsymbol{\Sigma}_C \tag{6}$$

$\boldsymbol{\Sigma}_T$ is the total covariance matrix, and $\boldsymbol{\Sigma}_A$, $\boldsymbol{\Sigma}_B$, and $\boldsymbol{\Sigma}_C$ are covariance explained by attributes A, B and C respectively. $\boldsymbol{\Sigma}_e$ is the residual variance that is not explained by these attributes.

Here,

$$\boldsymbol{m}_{...} = \frac{1}{abc}\sum_{i=1}^{a}\sum_{j=1}^{b}\sum_{k=1}^{c}\boldsymbol{m}_{ijk} \tag{7}$$

$$\boldsymbol{m}_{i..} = \frac{1}{bc}\sum_{j=1}^{b}\sum_{k=1}^{c}\boldsymbol{m}_{ijk} \tag{8}$$

$$\boldsymbol{m}_{.j.} = \frac{1}{ac}\sum_{i=1}^{a}\sum_{k=1}^{c}\boldsymbol{m}_{ijk} \tag{9}$$

$$\boldsymbol{m}_{..k} = \frac{1}{ab}\sum_{i=1}^{a}\sum_{j=1}^{b}\boldsymbol{m}_{ijk} \tag{10}$$

and

$$\boldsymbol{m}_{ijk} = \frac{1}{n_{ijk}} \sum_{l=1}^{n_{ijk}} \boldsymbol{x}_{ijkl} \tag{11}$$

Our objective was to find a projection that maximizes the variance of attribute A while minimizing the variances of attributes B and C. Specifically, we aimed to find $\boldsymbol{u}^*$ that maximizes the following equation:

$$\boldsymbol{u}^* = \arg\max_{\boldsymbol{u}} \frac{\boldsymbol{u}^T \mathbf{N}_A \boldsymbol{u}}{\boldsymbol{u}^T \mathbf{N}_e \boldsymbol{u}} \tag{12}$$

where

$$\mathbf{N}_A = \frac{1}{a-1}\boldsymbol{\Sigma}_A - \frac{1}{b-1}\boldsymbol{\Sigma}_B - \frac{1}{c-1}\boldsymbol{\Sigma}_C \tag{13}$$

and

$$\mathbf{N}_e = \frac{1}{N-a-b-c+2}\boldsymbol{\Sigma}_e \tag{14}$$

N is the total number of cells, and $a-1$, $b-1$, $c-1$, and $N-a-b-c+2$ are the degrees of freedom of the corresponding terms.

This optimization problem is commonly referred to as a generalized eigenvalue problem [2]. Here, $\mathbf{N}_A$ is symmetric but not necessarily positive definite, and $\mathbf{N}_e$ is positive definite. When $\mathbf{N}_e$ is invertible, the eigenvector $\boldsymbol{u}^*$ associated with the largest eigenvalue of $\mathbf{N}_e^{-1}\mathbf{N}_A$ is selected. In this study, we identify the eigenvector with the largest eigenvalue of $\mathbf{N}_e^{-1}\mathbf{N}_A$, which we refer to as the FLDA eigenvalue for the attribute A. This FLDA eigenvalue measures how much variance of the corresponding attribute (A) is captured compared to the variances of other attributes (B, C). The eigenvector $\boldsymbol{u}^*$ can be normalized to have a unit length. The elements within the unit vector represent the relative weights of the corresponding genes.

Similarly, to find a low-dimensional representation aligned with a categorical attribute B, we maximized the objective:

$$\boldsymbol{v}^* = \arg\max_{\boldsymbol{v}} \frac{\boldsymbol{v}^T \mathbf{N}_B \boldsymbol{v}}{\boldsymbol{v}^T \mathbf{N}_e \boldsymbol{v}} \tag{15}$$

where

$$\mathbf{N}_B = \frac{1}{b-1}\boldsymbol{\Sigma}_B - \frac{1}{a-1}\boldsymbol{\Sigma}_A - \frac{1}{c-1}\boldsymbol{\Sigma}_C \tag{16}$$

and to find a low-dimensional representation aligned with a categorical attribute C, we maximized the objective:

$$\boldsymbol{w}^* = \arg\max_{\boldsymbol{w}} \frac{\boldsymbol{w}^T \mathbf{N}_C \boldsymbol{w}}{\boldsymbol{w}^T \mathbf{N}_e \boldsymbol{w}} \tag{17}$$

where

$$\mathbf{N}_C = \frac{1}{c-1}\boldsymbol{\Sigma}_C - \frac{1}{a-1}\boldsymbol{\Sigma}_A - \frac{1}{b-1}\boldsymbol{\Sigma}_B \tag{18}$$

Implementation details

To examine the correspondence of primate parasol and midget RGCs with mouse α-RGCs, we utilized two published scRNA-seq datasets. The first dataset contains 35,699 adult RGCs, including 399 α-RGCs [6]. The second dataset contains macaque foveal RGCs containing 25,399 midget RGCs and 3306 parasol RGCs [4].

Data was preprocessed and normalized as described in refs. [6] and [4]. Briefly, transcript counts within each column of the count matrix (genes × cells) were normalized to sum to the median number of transcripts per cell, resulting in normalized counts Transcripts-per-million (TPM_{ij}) for gene i in cell j. We used a log-transformed expression matrix $E_{ij} = \ln(TPM_{ij} + 1)$ for further analysis.

We next identified 7779 high-variance genes (HVGs) using an approach that fits a relationship between the mean and coefficient of variation of gene expression [1, 3]. Next, we performed principal component analysis (PCA) on the dataset to remove multicollinearity. Finally, we analyzed the resulting PCs x cells matrix using FLDA [5].

In order to determine the mouse RGC types that best match the four predominant primate RGC types: ON/OFF midgets and ON/OFF parasols, we selected 20 candidates of mouse types with known polarity and kinetics based on previous studies (**Supplementary Table 4**). We drew all possible combinations of four RGC types from this set (n=432), and for each combination, we performed FLDA and calculated the eigenvalue corresponding to the polarity and the kinetics axes. We ranked these combinations based on their FLDA eigenvalues and identified the combination with the highest eigenvalue as the best match (**Fig. 5**).

References

[1] Chen, H.-I. H., Jin, Y., Huang, Y., and Chen, Y. (2016). Detection of high variability in gene expression from single-cell rna-seq profiling. *BMC genomics*, 17:119–128.

[2] Ghojogh, B., Karray, F., and Crowley, M. (2019). Eigenvalue and generalized eigenvalue problems: Tutorial. *arXiv preprint arXiv:1903.11240*.

[3] Pandey, S., Shekhar, K., Regev, A., and Schier, A. F. (2018). Comprehensive identification and spatial mapping of habenular neuronal types using single-cell rna-seq. *Current Biology*, 28(7):1052–1065.

[4] Peng, Y.-R., Shekhar, K., Yan, W., Herrmann, D., Sappington, A., Bryman, G. S., van Zyl, T., Do, M. T. H., Regev, A., and Sanes, J. R. (2019). Molecular Classification and Comparative Taxonomics of Foveal and Peripheral Cells in Primate Retina. *Cell*.

[5] Qiao, M. and Meister, M. (2020). Factorized linear discriminant analysis for phenotype-guided representation learning of neuronal gene expression data. *arXiv:2010.02171 [cs, q-bio]*.

[6] Tran, N. M., Shekhar, K., Whitney, I. E., Jacobi, A., Benhar, I., Hong, G., Yan, W., Adiconis, X., Arnold, M. E., Lee, J. M., Levin, J. Z., Lin, D., Wang, C., Lieber, C. M., Regev, A., He, Z., and Sanes, J. R. (2019). Single-Cell Profiles of Retinal Ganglion Cells Differing in Resilience to Injury Reveal Neuroprotective Genes. *Neuron*, 104(6):1039–1055.e12.

Supplementary Note 3: Geometric analysis of gene expression centroids to find mouse RGC types that optimally match primate midget and parasol RGC types

Source of data

The starting materials are the gene expression matrices for retinal ganglion cells published by Tran et al. [2] (mouse, 35,699 RGCs across all 45 types) and Peng et al. [1] (macaque, 28,849 MGCs and PGCs in fovea, 10,333 MGCs and PGCs in periphery). Note that MGCs are midget RGCs and PGCs are parasol RGCs. We merged the two data sets using a list of orthologous genes between mouse and macaque from Ensembl, which yielded $N_{\text{genes}} = 10,416$ common genes. The cells fall into 53 different types: the 45 mouse RGC types identified in Tran et al. [2] and the 8 MGC and PGC types in the foveal and peripheral region identified in Peng et al. [1].

Selection of genes

For each gene and each type, we computed the mean and SEM of the expression level across cells of that type. For each gene, we then computed the signal-to-noise ratio (SNR): this is the variance of expression across the 53 types divided by the variance within those types. Genes were selected for analysis in order of decreasing SNR. About 50% of the genes had SNR>13, and 10% had SNR>100. Results reported here are based on genes with SNR>200. Owing to the high SNR, the cluster center of each cell type is very well defined: The standard error of the mean is a small fraction of the separation between the centers. In subsequent analysis we therefore ignore the variation of gene expression within clusters, and focus entirely on the cluster centers.

Dimension reduction

The N_{types} cluster centers live in an $(N_{\text{types}} - 1)$-dimensional space. Dimensions outside of that subspace are uninformative, because the cluster centers don't vary along those dimensions. So one can project the data into this subspace without loss of information. This results in a $N_{\text{types}} \times N_{\text{features}}$ data matrix, where $N_{\text{types}} = 53$ and $N_{\text{features}} = N_{\text{types}} - 1 = 52$. To reduce the co-variation between these features, we further reduced the dimensionality with a PCA step to N_{dims} dimensions.

Model for the geometry of gene expression vectors

Say the 8 gene expression vectors in the reduced feature space are

$$\vec{x}_{i,j}, \quad i \in \{1, \ldots, L = 4\}, j \in \{1, \ldots, M = 2\}$$

where $i = 1, \ldots, 4$ indexes the cell type (Off-transient, Off-sustained, On-transient, On-sustained) and $j = 1, 2$ indexes the species (macaque, mouse).

The model fits these 8 vectors with:

$$\vec{y}_{i,j} = \vec{a} + \vec{b}_i + \vec{c}_j$$

The model parameters are the vectors $\vec{a}$, $\{\vec{b}_i\}$, $\{\vec{c}_j\}$. The $\{\vec{b}_i\}$ describe the shape of the geometric arrangement within each species, and the $\{\vec{c}_j\}$ account for the shift from one species to the other. For uniqueness we require that

$$\sum_i \vec{b}_i = \sum_j \vec{c}_j = 0$$

We want to minimize the squared residual

$$\chi^2 = \sum_{i,j} (\vec{x}_{i,j} - \vec{y}_{i,j})^2$$

which is achieved by the solution

$$\vec{a} = \langle \vec{x}_{i,j} \rangle_{i,j} = \frac{1}{LM} \sum_{i,j} \vec{x}_{i,j}$$

$$\vec{b}_i = \langle \vec{x}_{i,j} - \vec{a} \rangle_j = \frac{1}{M} \sum_{j} (\vec{x}_{i,j} - \vec{a})$$

$$\vec{c}_j = \langle \vec{x}_{i,j} - \vec{a} \rangle_i = \frac{1}{L} \sum_{i} (\vec{x}_{i,j} - \vec{a})$$

To score the quality of the fit, we measure how much of the variance in the data it explains within each species. The mean expression vector within each species is

$$\vec{a}_j = \langle \vec{x}_{i,j} \rangle_i = \frac{1}{L} \sum_{i} \vec{x}_{i,j}$$

and the intra-species variance of the data is

$$V = \left\langle (\vec{x}_{i,j} - \vec{a}_j)^2 \right\rangle_{i,j} = \frac{1}{LM} \sum_{i,j} (\vec{x}_{i,j} - \vec{a}_j)^2$$

The intra-species variance explained by the model is

$$V_{\text{exp}} = \left\langle (\vec{y}_{i,j} - \vec{a}_j)^2 \right\rangle_{i,j} = \frac{1}{L} \sum_{i} \vec{b}_i^2$$

The fit score reported in the figure is the fraction of explained variance, V_{exp}/V.

Analysis and robustness

In initial analyses, we matched every candidate combination of 4 mouse RGC types listed in **Supplementary Table 4** to the 4 types in the macaques. For each such combination we fit the geometric model and computed the fraction of explained variance within species, V_{exp}/V. Obtaining results consistent with FLDA, we then sought to evaluate the matching of all $\binom{45}{4}4! = 3,575,880$ combinations of 4 mouse RGC types. To ensure robustness of the results, we repeated the analysis for many settings of the two parameters: $N_{\text{genes}} = 100, 200, 400$ and $N_{\text{dims}} = 6, 12, 24$. The fraction of explained variance reported in the figures are an average over all these conditions.

References

[1] Peng, Y.-R., Shekhar, K., Yan, W., Herrmann, D., Sappington, A., Bryman, G. S., van Zyl, T., Do, M. T. H., Regev, A., and Sanes, J. R. (2019). Molecular Classification and Comparative Taxonomics of Foveal and Peripheral Cells in Primate Retina. *Cell*.

[2] Tran, N. M., Shekhar, K., Whitney, I. E., Jacobi, A., Benhar, I., Hong, G., Yan, W., Adiconis, X., Arnold, M. E., Lee, J. M., Levin, J. Z., Lin, D., Wang, C., Lieber, C. M., Regev, A., He, Z., and Sanes, J. R. (2019). Single-Cell Profiles of Retinal Ganglion Cells Differing in Resilience to Injury Reveal Neuroprotective Genes. *Neuron*, 104(6):1039–1055.e12.
