# Supplementary material for: Evolution of neuronal cell classes and types in the vertebrate retina
Source: Nature. 2023 Dec 13;624(7991):415–24. doi: 10.1038/s41586-023-06638-9 (PMC10719112; doi:10.1038/s41586-023-06638-9)
Supplement: Supplementary file 2 — Reporting Summary [file 41586_2023_6638_MOESM2_ESM.pdf]

nature portfolio

Corresponding author(s): Karthik Shekhar and Joshua R. Sanes

Last updated by author(s): Aug 24, 2023

Reporting Summary

Nature Portfolio wishes to improve the reproducibility of the work that we publish. This form provides structure for consistency and transparency in reporting. For further information on Nature Portfolio policies, see our Editorial Policies and the Editorial Policy Checklist.

Statistics

For all statistical analyses, confirm that the following items are present in the figure legend, table legend, main text, or Methods section.

n/a	Confirmed	
☐	☒	The exact sample size (*n*) for each experimental group/condition, given as a discrete number and unit of measurement
☐	☒	A statement on whether measurements were taken from distinct samples or whether the same sample was measured repeatedly
☐	☒	The statistical test(s) used AND whether they are one- or two-sided<br>*Only common tests should be described solely by name; describe more complex techniques in the Methods section.*
☒	☐	A description of all covariates tested
☐	☒	A description of any assumptions or corrections, such as tests of normality and adjustment for multiple comparisons
☐	☒	A full description of the statistical parameters including central tendency (e.g. means) or other basic estimates (e.g. regression coefficient) AND variation (e.g. standard deviation) or associated estimates of uncertainty (e.g. confidence intervals)
☐	☒	For null hypothesis testing, the test statistic (e.g. *F*, *t*, *r*) with confidence intervals, effect sizes, degrees of freedom and *P* value noted<br>*Give P values as exact values whenever suitable.*
☒	☐	For Bayesian analysis, information on the choice of priors and Markov chain Monte Carlo settings
☒	☐	For hierarchical and complex designs, identification of the appropriate level for tests and full reporting of outcomes
☒	☐	Estimates of effect sizes (e.g. Cohen's *d*, Pearson's *r*), indicating how they were calculated

Our web collection on statistics for biologists contains articles on many of the points above.

Software and code

Policy information about availability of computer code

Data collection	10X Chromium V3, Illumina NovaSeq 6000, Zeiss LSM 900 confocal microscopes with 405, 488, 568 and 647 nm lasers, and processed using Zeiss ZEN software suites.
Data analysis	For 10X Chromium V3 datasets, reads were aligned and gene expression was quantified using Cellranger v7.0 (https://support.10xgenomics.com/single-cell-gene-expression/software/downloads/latest). To include both exonic and intronic reads in gene expression, we applied velocyto v1.0 to the corresponding bam files from Cellranger alignment (http://velocyto.org). The downstream clustering and integration analysis was done in R using Seurat v4.3.0 (https://satijalab.org/seurat/) and other packages (MASS v7.3.60, pvclust v2.2.0, reshape2 v1.4.4, stats v4.3.0, ggplot2 v3.4.2, dendextend v1.17.1 and ggdendro v0.1.23). Custom R code written for these analyses for the paper is available via Zenodo (https://zenodo.org/record/8067826) and GitHub (https://github.com/shekharlab/RetinaEvolution). Analysis for Factorized linear discriminant analysis (FLDA) and Geometric analysis of gene expression (GAGE) was performed in Python and the codes are available at https://github.com/muqiao0626/FLDA and https://github.com/markusmeister/Gene-Geometry, respectively.<br>BD FACSDiva v8.0.2 was used for FACS sorting.

For manuscripts utilizing custom algorithms or software that are central to the research but not yet described in published literature, software must be made available to editors and reviewers. We strongly encourage code deposition in a community repository (e.g. GitHub). See the Nature Portfolio guidelines for submitting code & software for further information.

March 2021

Data

Policy information about availability of data

All manuscripts must include a data availability statement. This statement should provide the following information, where applicable:

- Accession codes, unique identifiers, or web links for publicly available datasets
- A description of any restrictions on data availability
- For clinical datasets or third party data, please ensure that the statement adheres to our policy

The raw and processed sequencing data produced in this work has been submitted to the Gene Expression Omnibus (GEO) and are available via the accession number GSE237215. The species specific datasets are available via the SubSeries accession numbers GSE237202-214. The data will be publicly released on Nov 1, 2023 or upon publication, whichever is earlier. Previously published data utilized in this paper was downloaded from GEO repositories with accession numbers GSE81905, GSE137400, GSE152842, GSE148077, GSE15910, and GSE236005. Species phylogenetic trees were downloaded from the UCSC Genome Browser database (https://genome.ucsc.edu), and species reference genomes are available on Ensembl (https://www.ensembl.org)

Field-specific reporting

Please select the one below that is the best fit for your research. If you are not sure, read the appropriate sections before making your selection.

☒ Life sciences ☐ Behavioural & social sciences ☐ Ecological, evolutionary & environmental sciences

For a reference copy of the document with all sections, see nature.com/documents/nr-reporting-summary-flat.pdf

Life sciences study design

All studies must disclose on these points even when the disclosure is negative.

Sample size	Sample sizes were not predetermined. The mouse and macaque data presented here is from prior studies (Shekhar et al., Cell, 2016; Tran et al., Neuron, 2019; Peng et al., Cell, 2019). For the case of humans, we supplemented an existing dataset (Yan et al.,Scientific Reports, 2020) with 2x more BCs and 7x more RGCs. For the remaing species, we obtained 2-5 biological replicates per species, and sample sizes were chosen to recover >8000 RGCs and >5000 BCs, numbers that we deemed sufficient based on downsampling analyses using the mouse data. We collected sufficient RGCs for all species except cow, sheep and lamprey, and sufficient BCs for all species except lamprey and brown-anole lizard. However, the low sample size is partially compensated during integration, where the analysis of several species together aids in resolving distinct cell types.
Data exclusions	For the purposes of cell class annotation, no data was excluded. Low quality nuclei that possessed few reads, clusters of cells that did not express canonical markers of any known cell class, and clusters that mapped to other cell types found at much lower frequency (e.g. endothelial cells, microglia) were annotated as "Other" and were not considered for further analysis.<br><br>For the purposes of annotating cell types within a class, we first subsetted the data by class. Cells with abnormally high (> mean 1277 + 2*SD) or low (< mean - 2*SD) counts were removed. We also removed replicate batches that contained the class of interest at a frequency less than 50 cells. These cells were then clustered to identify types. Some clusters were removed if their top DE markers were widely expressed in several clusters, if they had lower RNA counts compared to other clusters, or if several of the top DE markers were canonical markers for cell classes other than the class of interest.
Replication	Flow cytometry data were reproducible across the biological replicates used for each species and across different cell/nuclei isolations from individual tissue donors (e.g. fovea vs. periphery). >2-3 flow cytometry experiments were performed in each case to ascertain optimal parameters, and then independent experiments were performed for each sequencing channel obtained for each animal.<br><br>RNA-seq: The reproducibility of OrthoTypes (OTs) was assessed by repeating the integration and clustering analysis 50 times by repeated sampling of cells from the datasets.<br><br>Methods: The orthotype identification was repeated using four alternative methods : Seurat, Harmony, scVI and Liger. We found all methods to produce consistent results, although results in Bipolar Cells (where extensive ground truth is available) suggests that Seurat is superior.
Randomization	All species specimens were controls and were therefore allocated into the same experimental group. Randomization was not used.
Blinding	Samples of human origin were de-identified and assigned a unique numerical ID. Researchers responsible for data generation had access to basic information about donors (age, sex), as well as the unique numerical ID assigned to each donor.<br>For experiments other than those involving human specimens, similar donor metadata was available to researchers involved in data generation and analysis.<br>Blinding was not relevant to these experiments as all data were from control samples and moreover, knowledge of the species was needed for picking the correct reference genome/transcriptome for each species.

Reporting for specific materials, systems and methods

We require information from authors about some types of materials, experimental systems and methods used in many studies. Here, indicate whether each material, system or method listed is relevant to your study. If you are not sure if a list item applies to your research, read the appropriate section before selecting a response.

Materials & experimental systems

n/a	Involved in the study
☐	☒ Antibodies
☒	☐ Eukaryotic cell lines
☒	☐ Palaeontology and archaeology
☐	☒ Animals and other organisms
☐	☒ Human research participants
☒	☐ Clinical data
☒	☐ Dual use research of concern

Methods

n/a	Involved in the study
☒	☐ ChIP-seq
☐	☒ Flow cytometry
☒	☐ MRI-based neuroimaging

Antibodies

Antibodies used

RBPMS, PhosphoSolutions #1832-RBPMS,
CHX10, Novus Biologicals #NBP1-84476,
AP2A, DSHB #3B5

Validation

These antibodies have been used extensively by many groups, including ours. We used the following dilutions: 1:500 RBPMS; 1:400 CHX10; 1:50 AP2A.

RBPMS, PhosphoSolutions #1832-RBPMS; From the manufacturer's website: This is a synthetic peptide corresponding to amino acid residues from the N-terminal region of rat RBPMS, conjugated to keyhole limpet hemocyanin (KLH). It can detect RBPMS in Blind Mole, Guinea Pig, Humans, Monkey, Mouse, Rabbit, Rat, and Tree Shrew. The antibody is prepared from guinea pig serum by affinity purification via chromatography on an affinity column prepared with the N-terminal peptide used as antigen. It is specific for endogenous levels of the ~24 kDa RBPMS protein and for quality control Western blots performed on each lot.

NeuN-PE, Milli-Mark, clone A60, #FCMAB317PE: From the manufacturer's website: Clone A60 is validated for use in flow cytometry for the detection of NeuN. Quality is evaluated by flow cytometry using U251 cells. The immunogen is purified cell nuclei from the mouse brain. It can detect RBPMS in humans.

CHX10/VSX2, Novus Biologicals #NBP1-84476; From the manufacturer's website: This pan-Bipolar cell marker was developed against Recombinant Protein corresponding to amino acids in VSX2 protein. It is validated using Western Blot and Immunocytochemistry/ Immunofluorescence: CHX10 Antibody [NBP1-84476] - Analysis in control (vector only transfected HEK293T lysate) and VSX2 over-expression lysate (Co-expressed with a C-terminal myc-DDK tag (3.1 kDa) in mammalian HEK293T cells). It can detect VSX2 in mice.

AP2A, DSHB #3B5; From the manufacturer's website: This monoclonal antibody developed against the AP-2 alpha delta N165 (DNA-binding domain) protein. It is confirmed to react with multiple species including Chicken, Humans, Mice, and Zebrafish.

Chx10 Antibody (E-12), Santa Cruz Biotechnology, #sc-365519; From the manufacturer's website: Chx10 Antibody (E-12) is a mouse monoclonal IgG2a κ Chx10 antibody, cited in 23 publications, provided at 200 µg/ml specific for an epitope mapping between amino acids 37-64 near the N-terminus of Chx10 of human origin. It is recommended for the detection of Chx10 of mouse, rat, and human origin by WB, IP, IF, IHC(P), and ELISA; also reactive with additional species, including equine, canine, and porcine. It is validated using direct fluorescent western blot analysis of Chx10 expression in human brain tissue extract and immunoperoxidase staining of formalin-fixed, paraffin-embedded human fetal eye tissue.

Animals and other organisms

Policy information about studies involving animals; ARRIVE guidelines recommended for reporting animal research

Laboratory animals

For mice, C57Bl6 strain was used and for zebrafish Tg(vsx1:GFP) nns5 was used. For the other animals strain information is not available.

Data from the following species were collected in this paper: Zebrafish BCs (Danio rerio; n = 80-130 animals split across 15 biological replicates), Brown anole lizard (Anolis sagrei; n = 3 animals). Opossum (Monodelphis domestica, n=5 animals), Ferret (Mustela putoriusfuro, n= 2 animals), Pig (Sus domesticus, n=4 animals), Cow (Bos taurus, n=6 animals), Sheep (Ovis aries, n=6 animals), Thirteen-lined ground squirrel (Ictidomys tridecemlineatus, n=1 animal), Deer mouse (Peromyscus maniculatus bairdii, n=3 animals), Four-striped grass mouse (Rhabdomys pumilio, n=2 animals), Tree shrew (Tupaia belangeri chinensis, n=3 animals), Marmoset (Callithrix jacchus, n=2 animals). Data for mouse, sea lamprey, Zebrafish RGCs and Chick are from other studies.

Both adult males and adult females were used in approximately equal numbers for each species.

The following ages were used: ferrets were ~1 year old; marmosets were 2-7 years old; lampreys were adults at >3 years old; opossums were 1.4-2 years old; tree shrews were 1.5 - 2 years old; zebrafish were 4-6 months old; peromyscus, ground squirrels and rhabdomys were 2 months-8 months old; lizards were ~1 year old.

Adult Pig, cow, and sheep eyes were collected from an abattoir so we do not have information about their exact age.

Wild animals	No wild animals used in this study.
Field-collected samples	No field collected samples used in this study.
Ethics oversight	Pig, cow and sheep eyes were obtained, on average, 1 hour from death from an abattoir located in West Groton, Massachusetts. Other animal eyes were obtained from animal colonies maintained at Brandeis University (ferret), California Institute of Technology (tree shrew), Harvard University (ferret), MIT (marmoset), NIH (squirrel), University of Manchester, UK (Rhabdomys), University of Georgia (lizard), and University of California, Los Angeles (lamprey, opossum). Animal experiments conducted in the United States were approved by the Institutional Animal Care and Use Committees (IACUC) in each location. Rhabdomys tissue was collected in accordance with the Animals, Scientific Procedures Act of 1986 (United Kingdom) and approved by the University of Manchester ethical review committee.

Note that full information on the approval of the study protocol must also be provided in the manuscript.

Human research participants

Policy information about studies involving human research participants

Population characteristics	Human eyes were obtained post-mortem at a median of 6 hours from death either from the Massachusetts General Hospital (MGH) via the Rapid Autopsy Program or through the Steele Center for Translational Medicine at the John A. Moran Eye Center (SCTM), University of Utah. Acquisition and use of human tissue were approved by the Human Studies Committees of at Harvard (Dana Farber/Harvard Cancer Center Protocol No. 13-416) and University of Utah (Protocol IRB_00010201).<br><br>Data from male and female subjects were obtained in roughly equal number with an age range 50-77 years. Data from n=18 individuals is presented here.
Recruitment	Postmortem tissue specimens from males and females between 50-77 years of age with no known history of ocular disease ('control' cases) were considered for inclusion in this study of single-cell transcriptomes
Ethics oversight	Human eyes were obtained post-mortem at a median of 6 hours from death either from Massachusetts General Hospital (MGH) via the Rapid Autopsy Program or from The Lion's Eye Bank in Murray, Utah. Acquisition and use of post-mortem human tissue samples were approved by either the Institutional Review Board of the University of Utah (Protocol IRB_00010201), or the Human Study Subject Committees at Harvard (Dana Farber/Harvard Cancer Center Protocol No. 13-416), and were in compliance with the National Human Genome Research Institute (NHGRI) policies. All donors were confirmed to have no history or clinical evidence of ocular disease or intraocular surgery.

Note that full information on the approval of the study protocol must also be provided in the manuscript.

Flow Cytometry

Plots

Confirm that:

- [x] The axis labels state the marker and fluorochrome used (e.g. CD4-FITC).
- [x] The axis scales are clearly visible. Include numbers along axes only for bottom left plot of group (a 'group' is an analysis of identical markers).
- [x] All plots are contour plots with outliers or pseudocolor plots.
- [x] A numerical value for number of cells or percentage (with statistics) is provided.

Methodology

Sample preparation	Frozen retinal tissues were homogenized in a Dounce homogenizer in 1ml Tris-based lysis buffer with 0.1% NP-40. Nuclei were stained with NEUN/RBFOX3 and/or CHX10/VSX2, washed once, pelleted at 500g for 5min, resuspended in PBS/BSA and stained with DAPI. NEUN+ and/or CHX10+ DAPI+ (single) nuclei were collected using a flow cytometer.
Instrument	BD FACS Aria Cell Sorter
Software	BD FACSDiva 8.02
Cell population abundance	0.7-6% of nuclei with highest expression of NeuN were selected to enrich for retinal ganglion cells. Likewise, 4-10% of nuclei with highest expression of CHX10 were selected to enrich for bipolar cells.
Gating strategy	Nuclei were gated based on FSC-A and SSC-A to exclude derbies. Next, DAPI+ nuclei (single nuclei) were selected. Finally, NeuN+ and/or CHX10+ nuclei were selected. For some sample all DAPI+ nuclei were selected.

- [x] Tick this box to confirm that a figure exemplifying the gating strategy is provided in the Supplementary Information.
